# Nanotechnology-empowered combination therapy for rheumatoid arthritis: principles, strategies, and challenges

**DOI:** 10.1186/s12951-024-02670-7

**Published:** 2024-07-22

**Authors:** Shujing Ren, Yuhang Xu, Xingpeng Dong, Qingxin Mu, Xia Chen, Yanyan Yu, Gaoxing Su

**Affiliations:** 1 Department of Pharmacy, Affiliated Hospital 2 of Nantong University, Nantong, 226000 PR China; 2 School of Pharmacy, Nantong University, Nantong, 226000 PR China; 3 Department of Pharmaceutics, University of Washington, Seattle, WA 98195 USA

**Keywords:** Rheumatoid arthritis, Combination therapy, Co-delivery, Multifunctional nanomedicine, Multimodal therapy

## Abstract

Rheumatoid arthritis (RA) is an autoimmune disease with multifactorial etiology and intricate pathogenesis. In RA, repeated monotherapy is frequently associated with inadequate efficacy, drug resistance, and severe side effects. Therefore, a shift has occurred in clinical practice toward combination therapy. However, conventional combination therapy encounters several hindrances, including low selectivity to arthritic joints, short half-lives, and varying pharmacokinetics among coupled drugs. Emerging nanotechnology offers an incomparable opportunity for developing advanced combination therapy against RA. First, it allows for co-delivering multiple drugs with augmented physicochemical properties, targeted delivery capabilities, and controlled release profiles. Second, it enables therapeutic nanomaterials development, thereby expanding combination regimens to include multifunctional nanomedicines. Lastly, it facilitates the construction of all-in-one nanoplatforms assembled with multiple modalities, such as phototherapy, sonodynamic therapy, and imaging. Thus, nanotechnology offers a promising solution to the current bottleneck in both RA treatment and diagnosis. This review summarizes the rationale, advantages, and recent advances in nano-empowered combination therapy for RA. It also discusses safety considerations, drug–drug interactions, and the potential for clinical translation. Additionally, it provides design tips and an outlook on future developments in nano-empowered combination therapy. The objective of this review is to achieve a comprehensive understanding of the mechanisms underlying combination therapy for RA and unlock the maximum potential of nanotechnology, thereby facilitating the smooth transition of research findings from the laboratory to clinical practice.

## Introduction

Rheumatoid arthritis (RA) is a systemic autoimmune disease with patients exhibiting symmetrical and polyarticular inflammation, pain and swelling [1]. In the absence of an effective treatment, it can evolve in significant joint damage, disability, and work loss. RA is a substantial challenge to cure because of its multifactorial etiology and complex pathogenesis [2]. Early diagnosis and treatment of RA are crucial, while pharmaceutical therapy currently dominates clinical practice [3]. Although RA management has revolutionized over time, resulting in higher disease remission levels and better long-term outcomes. Great challenges still remain, and the focus now is on how to achieve precision medication and realize disease control most cost-effectively among different therapies.

Therapeutic agents used to treat RA can be divided into four categories: non-steroidal anti-inflammatory drugs (NSAIDs), glucocorticoids (GCs), disease-modifying antirheumatic drugs (DMARDs), and biological agents [4]. In addition, various herbs and their derived phytochemicals have significantly contributed to RA treatment, thereby benefiting it [5]. However, monotherapy may be insufficient for addressing complex and progressive pathological changes in RA, as it is generally aimed at solving or partially solving one drug target or symptom, which may result in poor prognosis. For example, methotrexate (MTX), the first-line DMARD, can improve and delay RA progression, but it cannot directly relieve pain and inflammation [6]. Likewise, NSAIDs and GCs alleviate inflammation and pain, but cannot delay disease progression [7]. Therefore, current RA treatment strategies are increasingly holistic and integrated. Combination therapy has been proposed for RA treatment and widely investigated in both laboratories and clinics, aiming to exhibit synergistic pharmacological mechanisms and non-overlapping toxicities [8–10]. Nevertheless, combination therapy in conventional formulations is associated with several limitations, such as lack of targeting ability, short half-lives, and varying pharmacokinetics of the drugs used in combination [11]. These limitations hamper the effectiveness and safety of combination therapy, and therefore, more advanced and intelligent techniques are being explored to maximize the advantages of combination therapy.

Nanotechnology has remarkable potential for overcoming conventional formulation-encountered challenges because of its unique physicochemical properties, such as nanometric size, high surface area to volume ratio, targeting properties, and stimuli-responsive drug release patterns [12]. Nanotechnology-empowered combination therapy not only involves the co-delivery of drug combinations to lesion sites, but also facilitates the combining of emerging therapeutic nanomaterials and therapy modalities. First, nanoformulations play a crucial role in improving the physicochemical properties of combination drug candidates, including solubility, loading efficiency, and bioavailability. Additionally, in combination therapy, multiple components are co-delivered with a precise targeting ability and in an on-demand drug release manner. Second, nanomaterials can exert therapeutic effects, which is more than a supporting role in RA therapy. Nanomedicines can exert anti-inflammatory and anti-arthritic properties, as well as dominate energy conversion therapies [13]. These properties render them favorable candidates for synergistic RA treatment when combined with therapeutic agents. Third, nano-based combination platforms offer great opportunities for multimodal therapy. Different modalities such as imaging [14], phototherapy [15], and sonodynamic therapy (SDT) [16] can be integrated into one nanoplatform, thereby achieving theranostic and amplifying therapeutic efficacy under the cooperation of each modality. Therefore, nano-empowered combination therapy holds great promise in improving RA treatment and diagnosis.

**Fig. 1:**
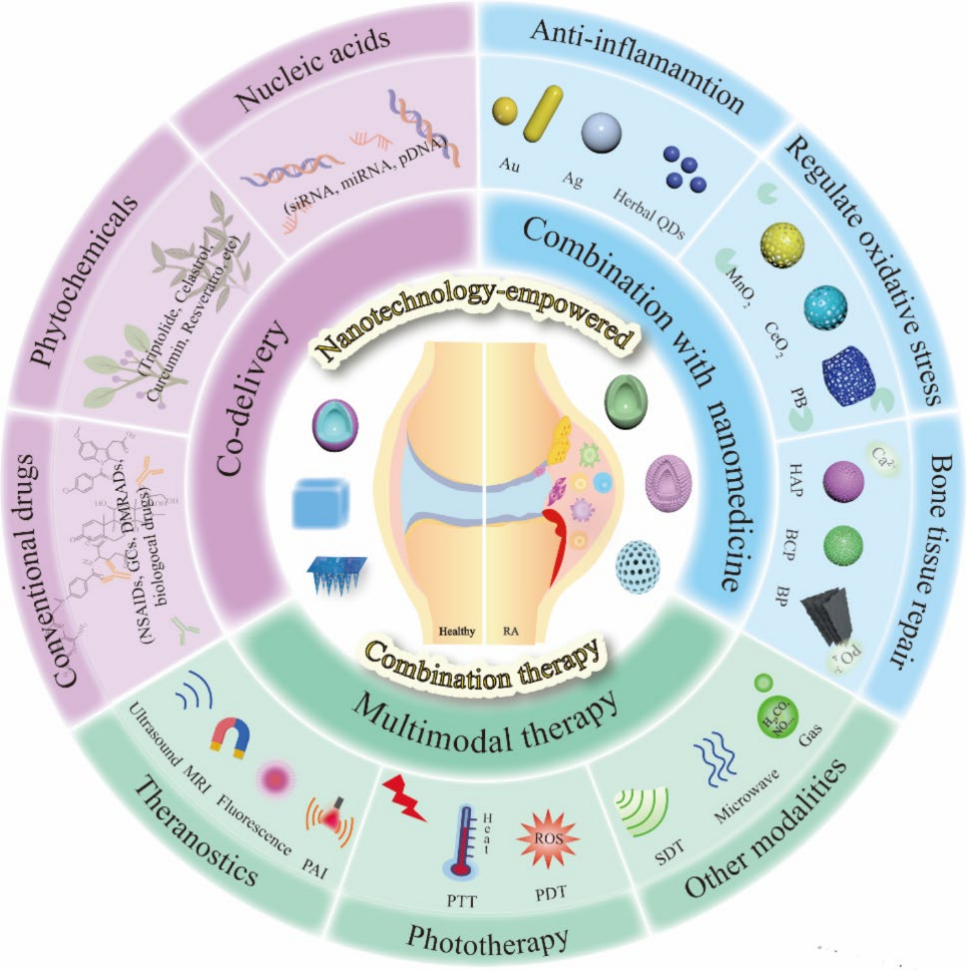
Schematic illustration of nanotechnology-empowered combination therapy and different combination strategies, including co-delivery of therapeutic agents, combination with therapeutic nanomaterials, and multimodal therapy

Considering that nanotechnology-empowered combination therapy is a promising and holistic strategy for RA, we thoroughly searched PubMed using key words such as combination therapy, nanotechnology, co-delivery, co-administration, and multimodal therapy, to explore recent progress in this field. This review offers an overview of the rationale for combination therapy; the benefits of nanotechnology; and diverse co-delivery strategies involving conventional drugs, phytochemicals, and nucleic acids. Furthermore, we discuss the combination with therapeutic nanomaterials and the facilitation of multimodal therapy (Fig. 1). Finally, we present challenges encountered in nanotechnology-empowered combination therapy from safety, drug-–drug interactions (DDIs), and clinical translation aspects, as well as future outlooks in designing intelligent and precise multifunctional nanoplatforms.

## The principle of combination therapy for RA

### RA pathogenesis

**Fig. 2:**
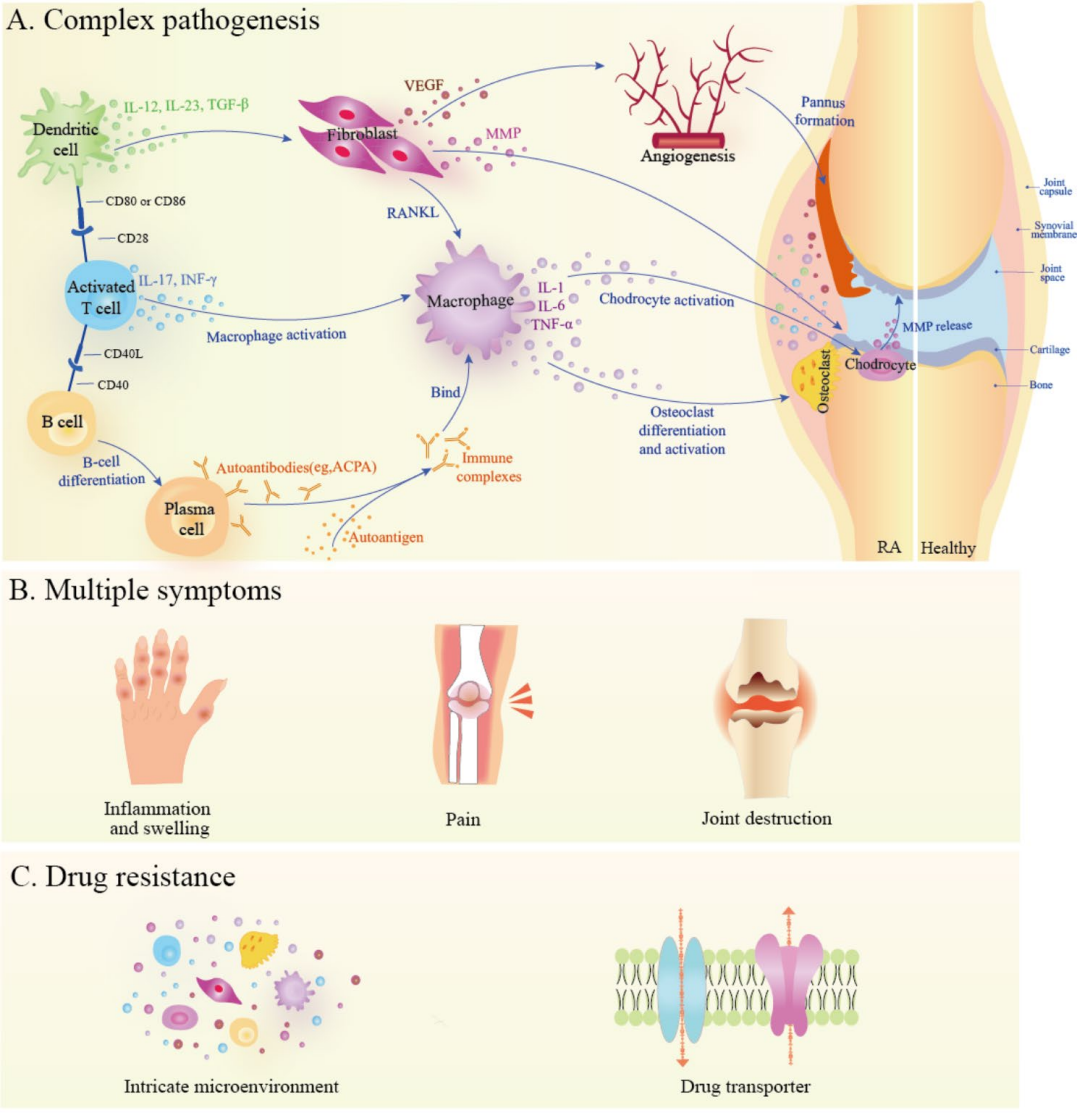
The principle of combination therapy for RA. (**A**) The coordinated interconnection of immune cells and non-immune cells. Autoreactive T-cells and B-cells promote the immune response against autoantigens. Typically, fibroblasts adopt proinflammatory and tissue-damaging functions by expressing MMPs and inflammatory mediators. Hypoxia and the production of vascular endothelial growth factor (VEGF) promote the formation of aggressive pannus tissues that can destroy the adjacent cartilage and bones. Moreover, activated macrophages produce pro-inflammatory cytokines and inflammatory infiltration activates osteoclast and chondrocytes, resulting in bone destruction and cartilage degradation. (**B**) Multiple symptoms of RA, including inflammation, swelling, pain, and joint destruction. (**C**) The main factors related to drug resistance in RA pharmacotherapy

The RA etiology remains incompletely understood. A multifactorial pathogenetic cascade involving genetic risk, environmental exposure, and microbiome interactions may drive RA development [17]. Dysregulated citrullination has been proposed to trigger the production of anti-citrullinated protein antibodies. This is manifested by indiscriminate attack of the body’s immune system, resulting in inflammation and joint capsule thickening, as well as promoting damage of adjacent bones and cartilage [18]. RA pathogenesis is intricate because of the coordinated interconnection between immune cells (e.g., T cells, B cells, dendritic cells, and macrophages) and non-immune cells (e.g., fibroblasts and endothelial cells) (Fig. 2A) [19]. This interplay involves the cross-linking of numerous signaling molecules playing specific roles in inflammatory processes, including Janus Kinase-Signal Transducer and Activator of Transcription (JAK-STAT), Mitogen-Activated Protein Kinase (MAPK), Wingless/Integrated (Wnt), and Notch signaling pathways [20]. Autoreactive T and B lymphocytes are considered the central drivers of the disease that promote the immune response against autoantigens. Activating fibroblast-like synoviocytes (FLS) and macrophage-like synoviocytes (MLS) then expands the intima, thereby creating a foundation for the adhesion and proliferation of inflammatory cytokines and proteases [21]. The MLS synthesizes pro-inflammatory cytokines such as IL-1, IL-6, and TNF-α. Simultaneously, FLS exhibit proinflammatory and tissue-damaging functions by expressing matrix metalloproteinases (MMPs) and inflammatory mediators, including prostaglandins and leukotrienes [22, 23]. Additionally, angiogenesis is a pivotal player as it facilitates the recruitment of white blood cells and synovial tissue hyperplasia, as well as leads to the formation of the destructive pannus tissue that harms neighboring cartilage and bones [24]. Overall, the intricate etiology and pathogenesis present great challenges to RA treatment. Gaining a deeper understanding of the underlying mechanisms and seeking versatile treatments for RA in a complementary manner is crucial.

### The rationale and clinical application of combination therapy

Because RA is so complex and refractory, the monotherapy efficacy is usually limited in clinics from many aspects. First, monotherapy cannot adequately address the intricate pathologies characterized by the crosstalk between numerous inflammatory cytokines, cells, and pathways (Fig. 2A). Second, RA is a chronic progressive disease with diverse symptoms such as inflammation, pain, and joint dysfunction (Fig. 2B) [25], monotherapy cannot handle multiple symptoms across the disease stages. Finally, RA requires long-term medication, but with monotherapy, drug resistance may occur due to the complex microenvironment, drug transporters, and other factors (Fig. 2C) [26, 27]. Combination therapy can be used to overcome these obstacles, wherein different drugs that complement or synergize with each other in their mechanisms are used simultaneously, thereby ensuring excellent overall efficacy. Moreover, each drug’s dosage can be lower-than-usual, which further reduces the occurrence of adverse reactions.

Combination therapy is an effective strategy for synergistically treating RA in terms of relieving diverse symptoms, reducing long-term joint damage, and preventing disability simultaneously [28]. According to the guideline, conventional synthetic DMARDs (csDMARDs) are the cornerstone in RA treatment, with MTX being the first-line choice. If MTX fails to achieve the treatment goal or is associated with severe adverse effects, treatment can be escalated by adding or substituting MTX with other csDMARDs (e.g., leflunomide and sulfasalazine), biological DMARDs (bDMARDs, e.g., tumor necrosis factor inhibitors, interleukin-6 inhibitors, and B-cell-depleting drugs), or targeted synthetic DMARDs (tsDMARDs, e.g., Janus kinase inhibitors) [29–31]. NSAIDs and GCs are typically included as adjunctive therapeutics for symptomatic management, thereby providing rapid relief of inflammation and pain. Numerous studies have compared the efficacy and safety of monotherapy and combination therapy in clinics. The combination of leflunomide and MTX was superior to MTX monotherapy in terms of clinical efficacy; complication incidence; alleviation of inflammatory markers, joint pain, and clinical symptoms, without any adverse effects [32]. In early-stage RA, the proportion of patients receiving adalimumab + MTX who achieved low disease activity, normal function, and radiographic non-progression at week 26 was significantly greater than that of patients starting on MTX monotherapy [33]. Along with chemical drugs and biological agents, Chinese medicine also plays a major role in combination therapy. For instance, several randomized control trials indicated that adding *Tripterygium wilfordii* Hook f. resulted in better efficacy than MTX monotherapy in RA patients, but caused no increase in the risk of adverse events [34]. Despite the augmented efficiency, combination therapy may also be associated with additional adverse events. According to a systematic review and meta-analysis, the combination of two bDMARDs increased the risk of side effects during the first 6–12 months of treatment, especially in RA patients receiving full doses [35]. Therefore, combination therapy has its pros and cons, and more efforts should be made to explore combination rules and mechanisms underlying them.

To sum up, combination therapy is effective in overcoming the limitations of monotherapy. However, the clinical outcome is not always satisfactory, because combination therapy may be associated with cumulative side effects resulting from the multiple agents added. Moreover, DDIs, administration sequence, and intervals are also key factors to be considered. Advanced combination therapy with the ability to maximize efficiency but minimize systemic side effects is desired.

## Advantages of nanotechnology in combination therapy

**Fig. 3:**
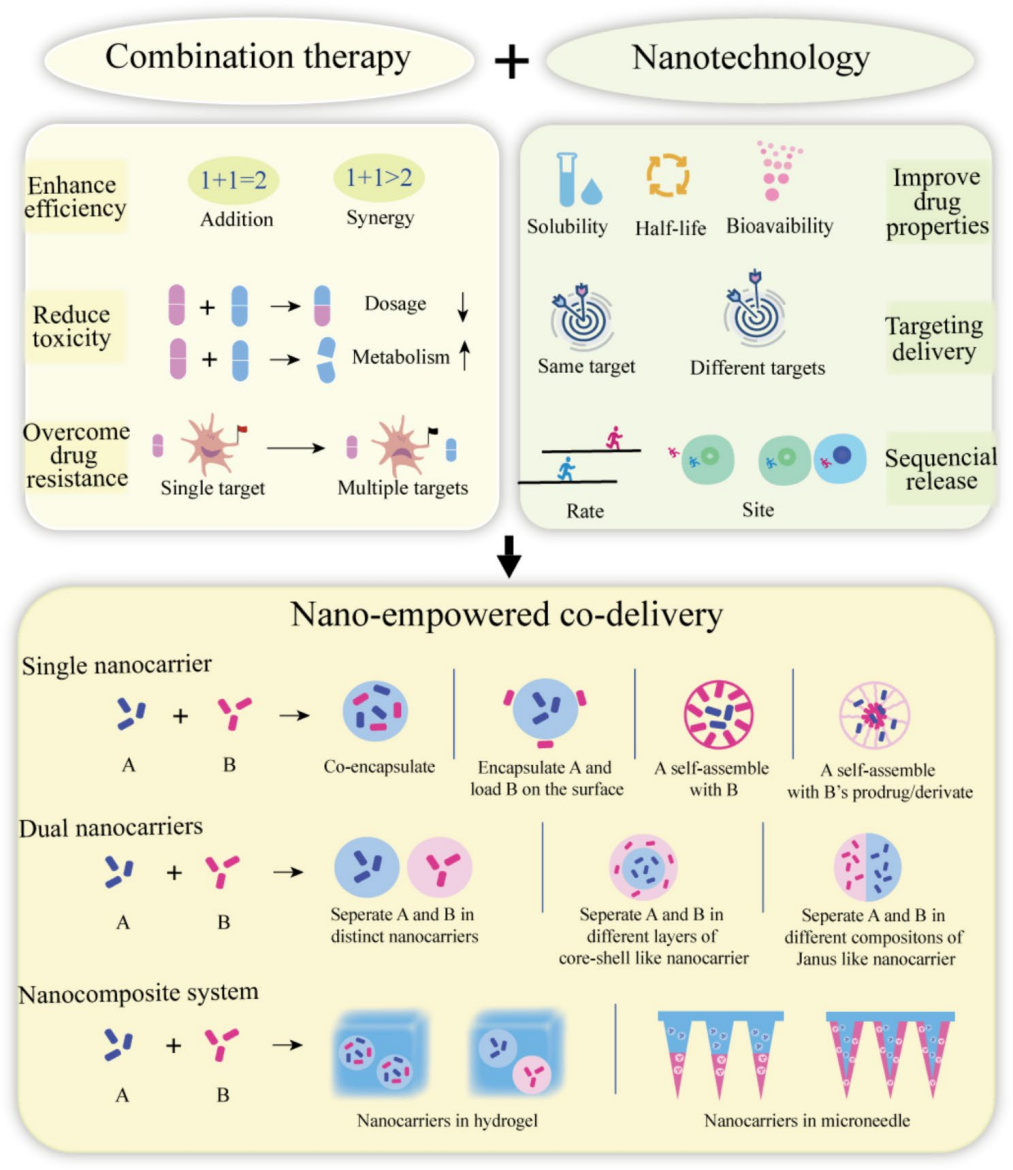
Rationale and strategies of nano-empowered co-delivery. Nano-empowered co-delivery offers the advantages of synergistic effects of multiple therapeutics with a well-designed target site and controllable release manner. Multiple therapeutics can be co-delivered by the same or separate nanocarriers based on their physicochemical properties and therapeutic requirements

Despite combination therapy being advantageous over monotherapy, co-administration of conventional dosage forms is suboptimal and unlikely to overcome all the monotherapy-associated problems because of several hindrances. Some of these hindrances are as follows: (a) the short elimination half-life and poor bioavailability of multiple drugs may subvert the success of combination therapy, (b) low selectivity in spatial distribution and therapeutic target leads to an inefficient efficacy and cumulative toxicity, and (c) the different pharmacokinetics and biodistribution of multiple drugs prevent them from achieving ideal stoichiometric ratio at the targeted site and exert synergistic anti-RA effects [36, 37]. Nowadays, the use of nanotechnology is flourishing rapidly in biomedical fields, thereby offering a great opportunity to revolutionize conventional combination therapy. We here summarize the benefits of nanotechnology as co-delivery systems, nanotherapeutics, and multifunctional nanoplatforms (Fig. 3).

### Improving drug properties

The efficacy of combination therapy in conventional formulations is low because of the poor aqueous solubility and oral bioavailability of many anti-arthritis drugs. Additionally, poor patient compliance and irregular administration, even self-withdrawal, are usually common in patients frequently receiving multiple drugs and for a long duration. When therapeutic agents are loaded in nanocarriers, the properties of nanoformulations determine their biological fates. First, nanoformulations can improve drug solubility by encapsulating the drugs in a hydrophobic cavity and thus increase the delivery efficiency in vivo. For instance, double-layered liposomes were designed to co-encapsule the hydrophobic drugs prednisolone and MTX in the inner core, and the outer layer was added to prevent premature leakage [38]. According to the results, the drug solubility was improved markedly, and the concentration of both drugs was higher in the inflamed joints than in the non-inflamed joints, thus finally exhibiting synergistic anti-inflammatory effects. Second, nanoformulations can extend the half-lives of co-delivered drugs and reduce the frequency and dosage of drugs to be administered. For instance, nimesulide and sulfasalazine were co-loaded into the halloysite lumen, followed by incorporation with a polymer hydrogel [39]. Different kinetic models (zero-order, first order, Higuchi, and Korsmeyer–Peppas) have been studied to suit single and combined drug release kinetics. The halloysite lumen and hydrogel decelerated the release rate of both drugs. The mechanisms of drugs released simultaneously differed from the release profiles on their own. When released independently, nimesulide exhibited no burst release. However, during co-delivery with sulfasalazine, nimesulide was rapidly released. Hence, considering DDIs is crucial when designing co-delivery systems, which may accelerate or decelerate the release of the coupled drugs.

### Targeting delivery

Although combination therapy is usually more productive than monotherapy by affecting multiple pathways, it is also associated with overlapping side effects. The lack of tissue specificity is the key obstacle to drug efficacy and safety. Targeted therapy is an imperative but overwhelming challenge for RA, especially in combination therapy. Multiple drugs can be co-delivered to inflamed joints through different targeting modes, namely passive targeting, active targeting, and stimuli-responsive release profiles. Passive targeting benefits from the development of endothelial gaps which termed as extravasation through leaky vasculature and subsequent inflammatory cell-mediated sequestration effect [40]. Several properties, such as size, surface chemistry, and shape, play crucial roles in drug biodistribution, pharmacokinetics, and uptake patterns by cells and therefore should be considered when deliver combination candidates passively. Active targeting takes advantage of ligands overexpressed in arthritis joints or related cells (e.g., macrophages, fibroblasts, and endothelial cells). For instance, MTX and ortho-vanillin were target delivered to macrophages by using β-glucan microspheres as the carrier, because most of their surface has 1,3-β-glucan groups. This carrier can expedite receptor-mediated phagocytic cellular uptake through dectin-1 or β-glucan receptors abundantly present on the surfaces of macrophages and dendritic cells [41]. A suitable ligand needs to be tailored for an effective target. Numerous targeting receptors, such as folate receptor, CD44, vasoactive intestinal peptide receptor, scavenger receptor, mannose receptor, and α_v_β_3_ integrin receptor, have been used for active targeting [42]. More specific and precise targeting strategies for each drug need to be explored. At last, differences in the microenvironment of healthy and arthritis-affected tissues offer possible targets for developing bio-responsive nanoformulations [43]. Various stimuli can trigger responsive drug release so as to reduce premature drug leakage from nano-systems before the drugs enter the inflamed joints. The commonly used strategies include an endogenous diseased microenvironment (e.g., pH, enzymes, and reactive oxygen species [ROS]) and external intervention (e.g., ultrasound, magnetic field, and light) [44–47]. The targeting mode governs the biodistribution and accumulation efficiency of multiple agents at the target site and influences their synergistic effects. Different from a single drug delivery system, combination therapy involves a great challenge of distinguishing varying targets of multiple drugs and tailoring corresponding targeting modes.

### Controlling sequential delivery

**Fig. 4:**
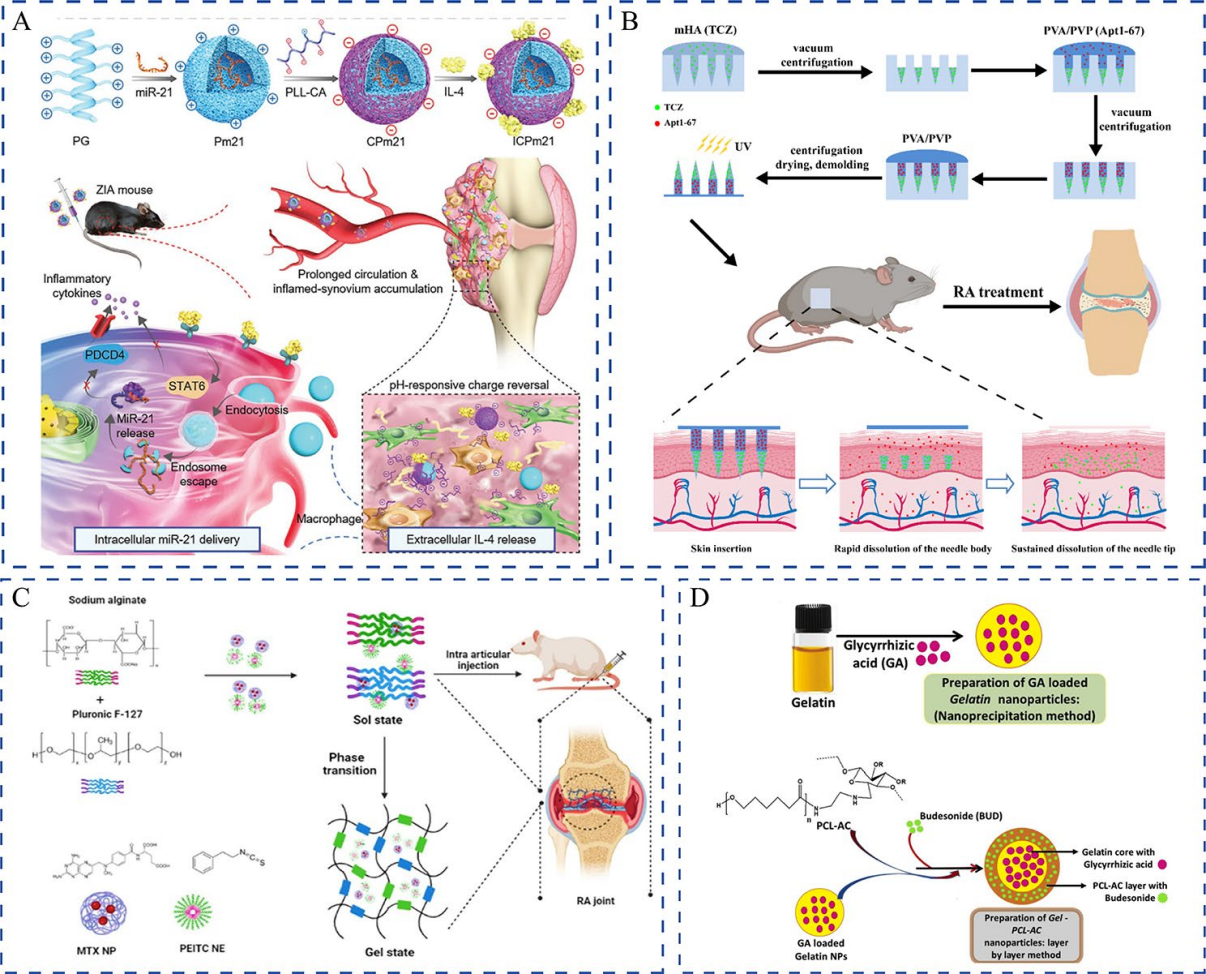
Strategies of co-delivering two therapeutic agents with distinct physiochemical properties and different targets. (**A**) Schematic illustration of the co-delivery of two therapeutics to different sites (intracellular/extracellular). Reproduced with permission copyright © 2021 WILEY-VCH [49]. (**B**) Schematic illustration of the co-delivery systems with different release rates of two therapeutic agents. Reproduced with permission copyright © 2022 MDPI AG [50]. (**C**) Schematic illustration of loading therapeutics by different nanocarriers and then incorporation into a hydrogel to prepare a nanocomposite hydrogel. Reproduced with permission copyright © 2023 Taylor & Francis [55]. (**D**) Schematic illustration of loading two therapeutic agents by nanocarriers with core-shell morphology. Reproduced with permission copyright © 2021 Elsevier [56]

Therapeutic agents often have varying pharmacokinetic characteristics. Nano-based co-delivery systems can control the temporal or spatial release of drugs and thus may achieve stronger combination effects than conventional co-administration [48]. For example, the pre-solving IL-4 must be liberated in the extracellular compartment of the inflamed synovium, whereas the anti-inflammatory microRNA-21 must be transported into the macrophage cytoplasm to induce effective transfection. In this scenario, Deng et al. proposed an intelligent strategy of sequential release at different extracellular/intracellular sites [49]. Nanocomplexes comprise a cationic inner core composed of the membrane-penetrating polypeptide (PG) and miR-21, and a charge reversal polymer (PLL-CA)-based outer layer with IL-4 adsorbed on the surface (Fig. 4A). In the acidic microenvironment of the inflamed synovium, PLL-CA transformed from negative to positive, which came off to liberate IL-4 extracellularly and facilitated the delivery of the PG/miR-21 core into macrophages. This strategy of sequentially releasing therapeutics at different extracellular/intracellular locations offers insights into precise combination therapy by controlling spatial release. Moreover, programmed release at different rates is also crucial, because it decides the order of treatment and is central to dosage-specific release of various therapeutics. An et al. designed a co-delivery system by combining tocilizumab and an aptamer Apt1-67 through separable microneedles [50]. The microneedles were designed for the sustained release of tocilizumab from the needle tips and rapid release of Apt1-67 from the needle bodies. Methacrylate-grafted hyaluronic acid and polyvinyl alcohol/polyvinyl pyrrolidone were used as the needle tips and needle bodies, respectively (Fig. 4B). Tocilizumab and Apt1-67 inhibit IL-6R and TNF receptor 1, respectively, and cooperate to improve the overall efficacy. In addition to microneedles, core-shell nanocarriers and nanocomposite hydrogels are also promising candidates for delivering anti-RA drugs sequentially [51–54]. Overall, site-specific localization and programmable release of therapeutic agents can markedly augment the exactitude of combination therapy and avoid undesirable interactions between multiple drugs.

### Developing new combination candidates and therapy modalities

Advancement in the spectrum of therapeutic tools and therapy modalities largely determines the treatment progress. Current pharmacotherapy is administered through conventional routes, such as orally, and requires long-term, high-dose, and frequent use, which results in several side effects [57]. Although nanotechnology has remarkably improved the drug delivery scenario, unlike a single drug delivery system, the efficiency of a co-delivery system may be limited by DDIs, the ratio of coupled therapeutics, and the premature leakage from nanocarriers. In this case, nanomaterials with therapeutic effects offer great advantages. Factually, nanomaterials are advantageous in theranostics for developing multifunctional nanomedicine [58]. Nanomaterials can be used for developing multimodal therapy through energy conversion, which integrates different therapy modalities, including phototherapy, SDT, and microwave-thermal therapy, into a single nanoplatform with complementary mechanisms [59, 60]. At last, nanomaterials with imaging, magnetic, or ultrasonic properties can serve as potent diagnostic platforms. Such platforms allow early RA diagnosis, guide personalized treatment, and enable evaluation of therapeutic effects. Compared with conventional combination therapy, nanotechnology-empowered combination therapy is more intelligent and versatile.

## Nano-empowered co-delivery of therapeutic agents

Co-delivery is widely used in combination therapy as it enhances efficacy and reduces non-overlapping toxicity by delivering two or more therapeutic agents simultaneously or sequentially. Nano-empowered co-delivery assimilates the advantages of combination therapy and nanotechnology, allowing the use of diverse and intelligent delivery strategies in combination therapy. To maximize efficacy and minimize side effects, it is crucial to have a precise understanding of the pharmacological mechanisms and interplay of each component, the balance of underlying competing elements, and synergistic treatment options. Then, nanocarriers can be accordingly tailored. The next inevitable question to be addressed is, “what would be the best combination regimen.” Multiple anti-RA drugs and permutations are available on the market, while clear guidelines for selecting the most appropriate combination strategies are lacking [61]. In this section, we illustrate different combination regimens based on conventional drugs, phytochemicals, and nucleic acids, thereby providing co-delivery strategies and revealing the action mechanisms underlying the co-delivered payloads.

### Co-delivery of conventional drugs

Conventional drugs, including DMARDs, NSAIDs, GCs, and biological agents, are the leading and widely used for RA treatment in clinics [62]. However, the clinical usefulness of these drugs is limited because of their low bioavailabilities and cumulative dose-related side effects. Although combination therapy can improve the efficacy through synergistic or complement pharmacological mechanisms, they may be associated with overlapping toxicity. Therefore, nano-based co-delivery systems have been broadly explored for the effective delivery of combination candidates.

GCs combined with DMARDs (typically MTX) are considered the first-line treatment for arthritis remission [63]. MTX and dexamethasone sodium phosphate (DSP) are frequently co-prescribed in clinics. However, they require separate, multiple dosing via oral and injectable routes, which lead to adverse reactions and influence patient compliance [64]. To reduce the amount and frequency of MTX and DSP to be administered, a phospholipid-based phase separation gel was developed to co-load the drugs and then injected locally into the inflamed joint cavity to achieve sustained efficacy [65]. The hydrogel offered sustained release of the dual drugs in a fixed ratio for 3 weeks. Thus, this system exhibited better anti-arthritis efficacy than free drugs. Unlike the hydrogel directly co-loaded with dual drugs, the nanocomposite hydrogel allows a more flexible design. In addition to the sustained release profile of the hydrogel, individual nanocarriers with different degradation behaviors can prevent unwanted drug interactions and control the release mode. To improve the therapeutic outcomes of leflunomide and dexamethasone, the drugs were pre-encapsulated in nanostructured lipid carriers and poly (lactic-co-glycolic acid) (PLGA) nanoparticles, respectively, followed by incorporation into a hydrogel to create a nanocomposite hydrogel [66]. Both nanoparticles exhibited a uniform particle shape with good entrapment efficiency. This nanocomposite hydrogel-based co-delivery system effectively prolonged the joint residence time, thereby promoting joint healing and suppressing RA progression.

NSAIDs are usually used as symptomatic drugs for relieving swelling and pian in RA. They cannot delay RA progression and therefore are mostly combined with DMARDs. Typically, indomethacin exerts immediate anti-inflammatory, analgesic, and detumescent effects, while increasing MTX absorption [67]. Thus, a local administration co-delivery system has been developed for MTX and indomethacin [68]. Bio-reducible polyethyleneimine was used for encapsulating dual drugs, and then, the obtained nanomedicines were loaded into a temperature-sensitive hydrogel comprising Pluronic F127 and Pluronic F68 for sustained drug release at the arthritis site. In a collagen-induced arthritis model, this well-designed co-delivery system significantly reduced inflammation and improved cartilage erosion because of the synergistic effect of MTX and indomethacin. Similarly, Wang et al. designed RGD peptide-mediated polymeric micelles to co-deliver the poorly water-soluble nimesulide and MTX that lacked tissue specificity [69]. The obtained micelles successfully loaded the drugs at a low dosage, and the drugs eventually exhibited potent synergistic effects in terms of inhibiting angiogenesis, suppressing inflammation, and deducing bone erosion. In addition to MTX, combination therapy with other csDMARDs has also been used. For instance, naproxen (NSAIDs) and sulfapyridine (DMARDs) were co-loaded in ethosomes and crosslinked with carbopol@934, thereby increasing the loading efficiency and prolonging the action time [70]. The entrapment efficiency of naproxen and sulfapyridine ethosomes was > 66%, and the ethosomes hydrogel exhibited a sustained release effect in effectively relieving pain and inhibiting inflammation. To improve patient compliance and reduce the systemic adverse effects of the drugs, Lu et al. constructed a transdermal dual drug delivery system, where dissolving microneedles and photothermal polydopamine were used to co-load tofacitinib (tsDMARDs) and loxoprofen (NSAIDs) [71]. According to the result, the drug combination synergistically downregulated inflammatory cytokines and reduced toxicity.

For patients who did not respond well to MTX monotherapy, combination therapy with another DMARD was recommended. Teriflunomide, an active leflunomide metabolite, was combined with MTX to overcome long-term and high-dose use-induced drug resistance and adverse effects [72]. Hydroxyapatite nanoparticles were employed as carriers owing to their good degradation in the acid arthritis environment and a chemical structure similar to that of bone materials. The co-delivery of MTX and teriflunomide successfully reduced the dosage to maximize the therapeutic efficacy and minimize the side effects by half. Furthermore, Pandey et al. constructed hyaluronic acid-coated hydroxyapatite nanoparticles to target CD44, thus actively delivering MTX and teriflunomide to arthritis joints [73]. According to pharmacodynamic studies, functionalized nanoparticles exhibit superior efficacy in preventing disease progression and promoting articular regeneration.

Various conventional drugs are co-delivered using well-designed nanocarriers for different therapeutic goals (Table 1). Nanocarriers can load these drugs through physical interaction, chemical link, or both. An appropriate drug group, identical pharmacokinetic profile, and ratiometric dose control are key parameters considered while using a single nanocarrier. If physicochemical properties are distinct, we can separate them into different compositions of a single nanocarrier or multiple carriers. Hybrid nanoparticles and nanocomposite platforms offer several advantages. Moreover, despite the potent improvement in efficacy and safety, additional work is required to verify whether the enhancement and reduction effects benefit from the simultaneously administered multiple drugs or the nanoplatforms used that could optimize the arthritis distribution and augment the retention of the coupled drugs.

**Table 1:** Co-delivery of conventional drugs

Drug 1	Drug 2	Co-delivery system	Adminis- tration	Mechanisms (downregulation [↓]/upregulation [↑])	Ref.
Methotrexate	Dexamethasone sodium phosphate	Phospholipid-based phase separation gel	Intra-articular	•↓TNF-α, IL-1β •Osteoprotect	[65]
Methotrexate	Prednisolone	Folate conjugated double layer liposomes	Intravenous	•Decrease paw edema	[38]
Leflunomide	Dexamethasone	Liposomes, PLGA nanoparticles and hydrogel	Intra-articular	•↓TNF-α •Promote joint healing	[66]
Methotrexate	Indomethacin	Bio-reducible polyethyleneimine nanoparticles and hydrogel	Intra-articular	•↓TNF-α, IL-6 •Improve cartilage erosion	[68]
Methotrexate	Nimesulide	Polymeric micelle	Intravenous	•↓TNF-α,IL-1β •Suppress angiogenesis • •Alleviate joint swelling •Reduce bone erosion	[69]
Sulfapyridine	Naproxen	Ethosomes and hydrogel	Transd-ermal delivery	•Decrease paw edema and limb circumference	[70]
Tofacitinib	Loxoprofen	Polydopa-mine NPs and microneedle	Transd-ermal delivery	•↓TNF-α, IL-1β, iNOS, JAK2, JAK3, and STAT3	[71]
Methotrexate	Teriflunomide	Hydroxya -patite nanoparticles	Subcutaneous	•Relieve inflammation •Improve articular structure regeneration •Reduce hepatotoxicity	[72]
Methotrexate	Teriflunomide	HA-coated h- ydroxyapatite nanoparticles	Intra-articular	•↓TNF-α, IL-1β,IL-6 •Promote articular regeneration •Reduce hepatotoxicity	[73]

### Co-delivery with phytochemicals

Nowadays, increasing attention has been paid to herbs and natural products, which serve as a vast resource for anti-arthritis agents [74]. Many phytochemicals exhibit high anti-arthritis activity with multiple targets and less adverse effects, such as triptolide, celastrol, curcumin, resveratrol, and quercetin. When combined, phytochemicals and chemotherapeutics or biologic agents are extremely promising in effective RA treatment (Table 2).

**Fig. 5:**
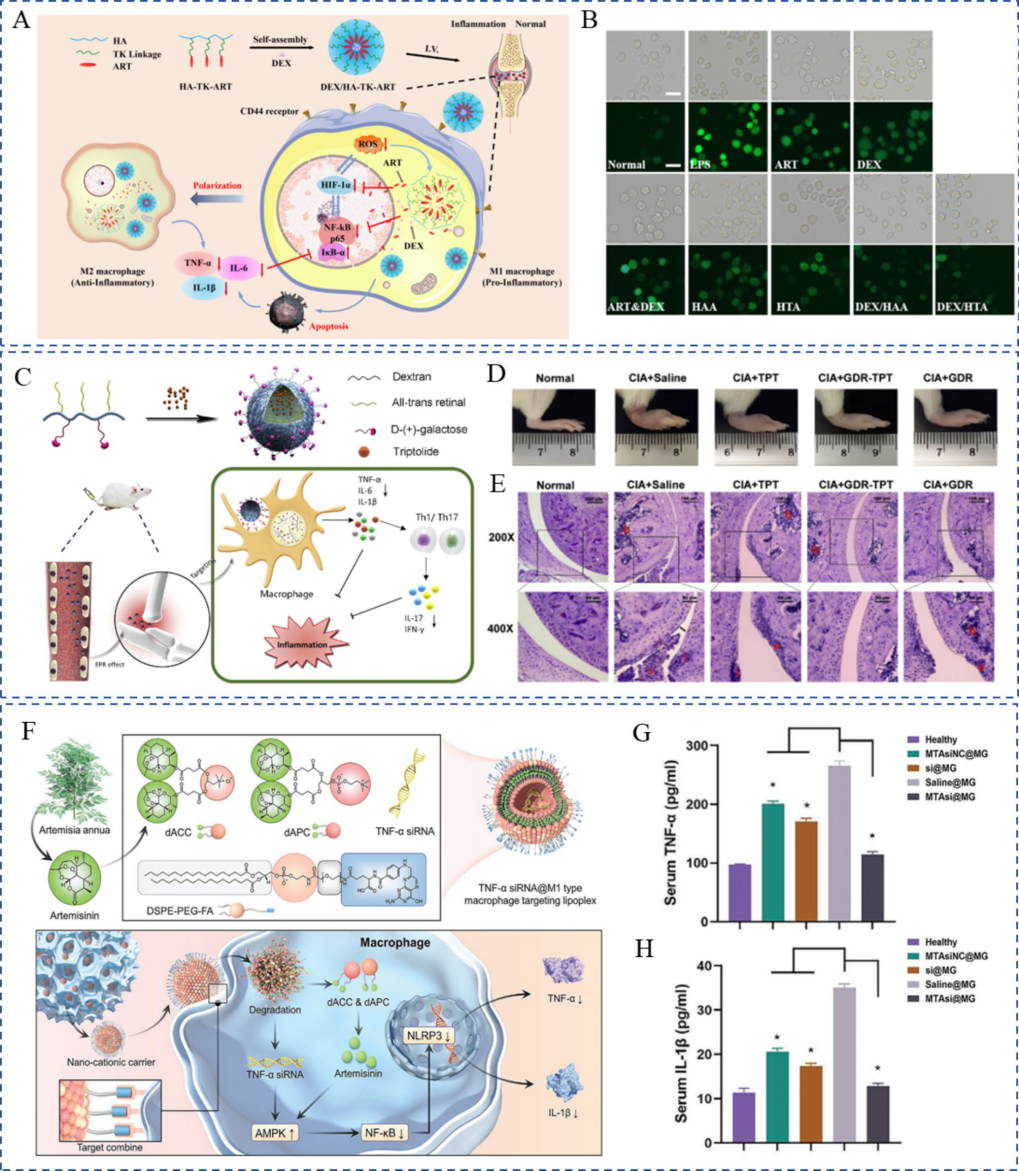
Different co-delivery systems based on the self-assembly strategy. (**A**) Schematic diagram of pH-responsive TP-loaded nanomedicine by self-assembly between artesunate-prodrug and dexamethasone. (**B**) The ROS-responsive co-released artesunate and dexamethasone exerted a synergistic action in scavenging ROS. Reproduced with permission copyright © 2022 Elsevier [80]. (**C**) Schematic illustration of the preparation of GDR-triptolide by self-assembly between all-trans-retinal derivates and triptolide. (**D**–**E**) GDR-improved anti-inflammatory effect of triptolide in a collagen-induced model. Reproduced with permission copyright © 2020 Elsevier [81]. (**F**) Schematic illustration showing the self-assembly of TNF-α siRNA-loaded dACC/dAPC-DSPE-PEG-FA lipoplexes. (**G**) Quantification of TNF-α and (H) IL-1β in the sample-treated RA rats serum specimens. Reproduced with permission copyright © 2022 WILEY-VCH [82]

Phytochemicals can function synergistically with chemotherapeutics to improve efficiency and decrease toxicity. For instance, to reduce the adverse effect and dosage of prednisolone, Yan et al. constructed a human serum albumin (HSA)-based nanoplatform. In this system, curcumin served as an adjunct therapy that improved the therapeutic efficacy of prednisolone and prevented arthritis development [75]. In adjuvant-induced arthritis rats, HSA-based co-delivery nanoparticles preferentially accumulated in inflamed joints and exhibited higher therapeutic efficacy than single-loaded nanoparticles or a simple mixture of curcumin and prednisolone. Similarly, MTX and curcumin were co-delivered using bovine serum albumin (BSA) to maximize the therapeutic efficacy and alleviate MTX-associated side effects [76]. On examining pharmacokinetics and pharmacodynamics, the study found that the BSA-MTX-curcumin nanoparticles had superior disease-modifying effects compared to free MTX. In addition to dosage reduction, regulating metabolism is another means of reducing side effects. For example, paeoniflorin combined with leflunomide offered synergistic pharmacodynamics in improving clearance and reducing liver injury [77]. However, such combination regimens have not yet been co-delivered, possibly because nanoparticles cannot reproduce this effect in dynamic transport. Apart from curcumin, resveratrol, a natural polyphenol with potent anti-inflammatory and antioxidant abilities [78], has been co-delivered to overcome the bioavailability issues and adverse effects of MTX monotherapy. The anti-arthritis activity of a transdermal gel containing resveratrol-MTX-loaded nanoemulsions has been assessed [79]. The co-delivered resveratrol and MTX inhibited inflammation and produced successful therapeutic options for RA. Similarly, Haloi et al. developed an MTX and phenethyl isothiocyanate (PEITC)-based nanocomposite hydrogel [55]. PETIC is a bioactive phytochemical found in many cruciferous vegetables. In this system, the two drugs were separated in different nanocarriers, PLGA nanoparticles, and nanoemulsions, respectively, and incorporated into the hydrogel for intra-articular injection (Fig. 4C). This nanocomposite hydrogel exhibited good anti-inflammatory activity and reversed cartilage disruption because of the synergistically functioning nanoparticulate forms of MTX and PEITC, thereby effectively improving the drawbacks of their free forms. However, the coupled drugs may sometimes have different hydrophilic and hydrophobic properties. In such cases, in addition to separating the drugs into two nanocarriers, nanoformulations comprising two distinct portions may be an ideal choice. Budesonide has been extensively used to alleviate inflammatory symptoms. Glycyrrhizic acid (GA), a natural triterpene glycoside from licorice roots, inhibited cyclooxygenase-2 (COX-2) and exerted potent anti-inflammatory effects. However, the oral bioavailability of budesonide and GA is poor owing to extensive drug metabolism and rapid clearance. Therefore, core-shell nanocarriers co-loaded with GA and budesonide have been designed [56]. Ansari et al. synthesized GA-loaded gelatin nanoparticles and coated them with budesonide-encapsulating aminocellulose-grafted polycaprolactone (Fig. 4D). In collagen-induced arthritis mice, this core-shell nanoplatform alleviated inflammation in terms of inflammatory cell infiltration, and biochemical and histological parameters. In addition to co-encapsulating drugs using external nanocarriers, the self-assembly between polymer prodrugs or free drugs extensively increased the loading capacity and realized stimuli-responsive release. To simultaneously deliver drugs to the inflamed joints and achieve microenvironment-responsive release, Li et al. cleverly tailored ROS-responsive artesunate prodrug micelles by linking artesunate and hyaluronic acid with a thioketal linker and then self-assembled to encapsulate dexamethasone (Fig. 5A) [80]. In this well-designed co-delivery system, artesunate and dexamethasone were circulated in the blood for a long time and accumulated in adequate amounts at the inflamed joints. This finally resulted in the synergistic cascade regulation of the HIF-1α/nuclear factor kappa-B (NF-κB) pathway, leading to ROS scavenging and macrophage repolarization (Fig. 5B).

In addition to inflammation and cartilage disruption, pain is another key reason for RA patients seeking medical help. To simultaneously treat inflammation and pain and refrain from long-term oral drug administration-induced toxicity, Kang et al. synthesized a nanostructured lipid carrier loaded with indomethacin and celastrol, a typical bioactive component of *T. wilfordii* Hook f. with potent anti-inflammatory and anti-arthritis properties [83]. Subsequently, the developed dual-drug nanomedicines were delivered transdermally through incorporation into Carbopol 940. Celastrol exerted anti-inflammatory activity and indomethacin acted as an analgesic agent. In vivo studies have demonstrated that this transdermal co-delivery system was prominent in decreasing paw edema and inhibiting inflammation and pain, without exhibiting any renal and reproductive toxicity. Likewise, to simultaneously relieve pain and suppress inflammation by using one nanoplatform, our group constructed an acupoint nanocomposite hydrogel. This hydrogel could preferentially deliver triptolide, an anti-RA agent, to the inflamed joints, while co-deliver 2-chloro-N (6)-cyclopentyl adenosine (CCPA) locally at the Zusanli acupoint to simulate acupuncture, thereby relieving pain [84]. In this novel co-delivery system, CCPA stimulated the acupoint for analgesic effects instead of directly delivering analgesics, thereby offering a promising strategy to bridge acupuncture and drug administration.

It is worth mentioning that phytochemicals are not always used as complementary or adjunctive therapeutics. Combination therapy and constructing co-delivery systems are also strategies for overcoming the limitations of phytochemicals themselves. Typically, triptolide exhibits potent anti-inflammatory and immunosuppressive activities, but its clinical application is restricted because of its multiorgan toxicity and poor solubility [85]. To resolve this issue, a novel co-delivery system comprising L-ascorbate palmitate (VP) and triptolide was created to synergistically treat arthritis and inhibit oxidative stress [86]. Notably, VP is a derivative of vitamin C, an effective antioxidant that inhibits triptolide-induced renal toxicity by regulating oxidative stress. Moreover, as a carrier, VP can encapsulate triptolide through the thin-film method along with cholesterol. The combination of VP and triptolide is a promising strategy for augmenting the water solubility and reducing triptolide’s toxicity. This study provided an ideal method for developing co-delivery systems by self-assembly between derivatives and therapeutics. Similarly, pH-sensitive galactosyl dextran-retinal (GDR) conjugates were synthesized and self-assembled with triptolide to improve its inflammatory effects, while reducing its systemic toxicity (Fig. 5C) [81]. Intriguingly, all-trans-retinal is GDR’s active component that is oxidized to generate retinoic acid. The retinoic acid reportedly inhibited inflammatory responses in RA. As expected, the GDR nanoparticles preferentially accumulated in inflammatory tissues, and the anti-arthritis effect of triptolide was markedly augmented (Fig. 5D, E), while its toxicity was attenuated through GDR-mediated encapsulation. Apart from modifying one active ingredient to encapsulate another, the self-assembly between prodrugs can be a promising strategy for realizing the responsive release of multiple components. Mixed micelles were developed through self-assembly between two prodrugs endowed with MMP-2 and ROS-responsive properties, respectively [87]. The curcumin prodrug was designed using the achyranthes polysaccharide as the hydrophilic end and the ROS-responsive thioketal as the bond. The celastrol prodrug was synthesized by linking dextran sulfate with celastrol containing the MMP-cleavage peptide PVGLIG. The resulting micelles had an average size of 204.8 nm and effectively treated RA with good safety.

Overall, numerous co-delivery systems have been developed for exploring the significance of phytochemicals in combination therapy. Compatibility is a principle of Chinese medicine, which can also be applied to phytochemical-based combination therapy to form the rules of Jun, Chen, Zuo, and Shi. However, co-delivering Chinese medicine formulations under current nanotechnology techniques is difficult because of their complex ingredients. More efforts are required to overcome technical difficulties and elucidate the action mechanism underlying combination regimens.

**Table 2:** Co-delivery with phytochemicals

Phyto- chemical	Other agents	Co-delivery system	Adminis- tration	Mechanisms (downregulation [↓]/upregulation [↑] )	Ref.
Curcumin	Prednisolone	HSA nanoparticles	Intravenous	•↓TNF-α, IL-1β, IL-6 •↑IL-10 •Reduce the dose of prednisolone	[75]
Curcumin	Methotrexate	BSA Nanoparticles	Intravenous	•Reduce swelling and paw edema •reduce the toxicity of MTX by curcumin-meditated oxidative stress inhibition	[76]
Resveratrol	Methotrexate	Nanoemulsion and hydrogel	Transdermal delivery	•Reduce swelling and paw edema	[79]
Phenethyl isothiocyanate	Methotrexate	PLGA nanoparticle, nanoemulsion and hydrogel	Intra-articular	•↓TNF-α, IL-1β, IL-6, IL-17 A, RANKL •↑IL-10	[55]
Glycyrrhizic acid	Budesonide	Gelatin- PCL-AC core-shell nanoparticles	Intravenous	•↓TNF-α, IL-1β, COX-2, iNOS •Ameliorate bone erosion	[56]
Artesunate	Dexameth-asone	Self-assembly nanomicelles	Intravenous	•↓TNF-α,IL-1β,IL-6, •Inhibit HIF-1α/NF-κB cascade •Scavenge ROS •Repolarize macrophage	[80]
Celastrol	Indomethacin	Liposomes and hydrogel	Transdermal delivery	•↓IL-1β, TNF-a, b-endorphin, substance P •Relieve pain	[83]
Celastrol	Curcumin	Self-assembly nanoparticles	Intravenous	Scavenge ROS	[87]
Triptolide	2-Chloro-N (6)-cyclopentyl adenosine	HSA nanoparticles and hydrogel	Acupoint administration	•Relieve pain •↓TNF-α,IL-1β,IL-6,IL-17 A ↑IL-10 •RebalanceTh17/Treg	[84]
Triptolide	L-ascorbate palmitate	VP self-assembly nanoparticles	Intravenous	•↓TNF-α,IL-1β, IL-6 •Alleviate toxicity of TP	[86]
Triptolide	Galactosyl dextran-retinal	GDR self-assembly Nanoparticles	Intravenous	•↓TNF-α, IL-1β, IL-6,IFN-γ,IL-17 A •↓Th1,Th17 •Alleviate toxicity of TP	[46]
Triptolide	miR-30-5p inhibitor	Mesoporous silica nanoparticles	Intravenous	•Inhibit fibroblast proliferation and increase apoptosis	[88]

### Co-delivery with nucleic acids

Nucleic acid-based therapeutics can interact with complementary nucleic acids inside cells and are used for targeted gene modulation. They possess lower toxicity than chemical drugs. These nucleic acids include small interfering RNA (siRNA), microRNA (miRNA), plasmid DNA (pDNA), messenger RNA (mRNA), and the CRISPR/Cas9 system [89]. However, nucleic acids are usually unstable and easily degradable. Moreover, negative charges and high molecular weights further hinder their efficient cellular uptake or lysosomal escape [90]. These limitations can be sufficiently overcome with nanotechnology. It also improves gene transfection efficiency. Nucleic acid-based co-delivery systems can exert synergistic effects by combining gene and chemical aspects (Table 3).

siRNAs have attracted substantial interest as they can specifically, reversibly, and flexibly silence RA pathogenesis-related target genes [91]. TNF-α is a critical player in RA inflammation. It is an autocrine stimulator and a strong paracrine inducer of some other inflammatory cytokines. Considering that TNF-α and overproduced ROS promote the RA pathological process, Li et al. co-delivered anti-TNF-α siRNA and alpha-tocopheryl succinate (α-TOS) to silence the TNF-α gene and decrease ROS levels of M1 macrophages, respectively [92]. α-TOS was covalently conjugated to the generation 5 poly(amidoamine) (G5 PAMAM) dendrimer to improve its solubility and bioavailability. Using the functionalized G5 PAMAM dendrimers as templates, Au nanoparticles were entrapped, which served as a vector for compacting TNF-α siRNA. In an in vivo investigation, this creative gene/drug co-delivery nanoplatforms was very promising for combined anti-inflammatory and antioxidative RA therapy. Alternatively, the TNF-α siRNA and artemisinin combination that exhibits potent anti-inflammatory and immunoregulatory properties has been proposed for RA treatment. However, TNF-α siRNA and artemisinin have extremely short half-lives in vivo. Hence, to co-encapsule TNF-α siRNA, Li et al. smartly designed a cationic multicomponent drug-embedded liposome based on two novel artemisinin derivatives (Fig. 5F) [82]. The liposomes were immobilized on porous microfluidic hyaluronic acid to achieve long-term treatment following injection into the joint cavity. As expected, the obtained TNF-α siRNA/artemisinin nano-micrcoplex successfully reduced inflammatory cytokine secretion (Fig. 5G, H), and inhibited synovial connective tissue hyperplasia and macrophage infiltration. Along with inflammatory cytokines, RA occurrence and progression-related pathways could also be used as potential targets. Activation of the NF-κB signaling pathway can induce the secretion of various inflammatory cytokines, which would, in turn, activate NF-κB and amplify the RA inflammatory response [93]. Accordingly, Wang et al. used p65 siRNA to inhibit the NF-κB signaling pathway by targeting the NF-κB family member p65. When further combined with dexamethasone, p65 siRNA inhibited NF-κB transcription and suppressed inflammation [94]. Polymeric hybrid micelles were used as co-delivery carriers, and this co-delivery of p65 siRNA and dexamethasone inhibited NF-κB signaling in macrophages and switched macrophages from the M1 to M2 state, which was more efficiently than micelles containing either of them alone. Duan et al. successfully co-delivered p65 siRNA and MTX by using folate-modified calcium phosphate/liposome-based hybrid nanocarriers [95]. The obtained nanomedicine effectively blocked the NF-κB signaling pathway and reduced the expression of pro-inflammatory cytokines, thereby offering the unique benefits of therapeutic efficacy and safety. Notch-1 is involved in TNF-α-induced proliferation in RA conditions, and the Notch-1 signaling pathway is involved in VEGF-based angiogenesis and hypoxia [96]. Inhibiting Notch-1 receptors using siRNAs could be an effective therapeutic strategy against RA. Zhao et al. successfully developed polymeric micelles co-loaded with MTX and Notch-1 siRNA. They demonstrated that the sequence-specific downregulation of Notch-1 expression augmented the anti-inflammatory effect, which served as an effective strategy for overcoming the limitations of both small molecules and siRNA [97]. Myeloid cell leukemia-1 (Mcl-1), a member of the anti-apoptotic B-cell lymphoma-2 protein family, is essential for blocking the apoptosis of the RA progression-promoting activated macrophages [98]. Mcl-1 siRNA can specifically silence Mcl-1 expression in activated macrophages to inhibit RA progression [99]. To improve the target efficacy of Mcl-1 siRNA and reduce the side effects of dexamethasone, Li et al. co-delivered Mcl-1 siRNA and dexamethasone by using designed folate-conjugated polymeric micelles. In this system, Mcl-1 mRNA expression in macrophage cells was significantly reduced and the superior anti-inflammatory property was noted [100]. Likewise, Li et al. used HA-coated pH-responsive nanoparticles to intelligently co-deliver Mcl-1 siRNA and dexamethasone to lesion sites [101]. The combination of Mcl-1 siRNA and dexamethasone synergistically promoted the apoptosis of activated macrophages and reduced the side effects of dexamethasone. Furthermore, Li and coworkers expanded on this work by co-delivering Mcl-1 siRNA, the hypoxia-activated prodrug tirapazamine, and the photothermal agent PCPDTBT [102]. They first synthesized mesoporous silica nanoparticles hybridized with PCPDTBT (HNs). Then, branched polyethyleneimine-folic acid was coated on the surface through electrostatic interactions, and tirapazamine was loaded into the mescopores and bound Mcl-1 siRNA on the surface. This multifunctional platform effectively silenced the target anti-apoptotic protein through Mcl-1 siRNA transfection. It also synergistically killed the activated macrophages through tirapazamine-meditated chemotherapy and NIR-induced phototherapy. In addition to regulating inflammatory pathways, siRNA can reverse cartilage and bone destruction. By developing a multifunctional drug delivery system composed of indomethacin, MTX, and MMP-9 siRNA, Yin et al. attempted to comprehensively treat RA through multiple signaling pathways [103]. Indomethacin immediately relieved pain and swelling in joints, while MTX slowed RA progression. MMP-9 siRNA addition reversed articular cartilage and bone disruption, and to some extent, reduced inflammatory cytokine release. Disulfide-crosslinked polyethyleneimine was synthesized to load indomethacin and MTX by using the solvent change method, and then incubated with MMP-9 siRNA to construct a nanomedicine containing triple drugs, which were further incorporated into hydrogel for in situ injection. This work provided a successful strategy for synergistically treating RA and other metabolic bone diseases.

miRNAs, which are highly conserved sequences and post-transcriptional regulators of gene expression, are emerging as a novel therapeutic modality [104]. Imperfect complementarity binding allows for the multi-targeting effect typical of miRNAs [105]. miRNA-124 (miR-124) is a potential therapeutic target against RA. Hao et al. designed self-assembly hybrid micelles to co-load miRNA and MTX and achieve their efficient delivery with low toxicity [106]. MTX was attached to linoleic acid-modified branched PEI (MTX-PEI-LA) and self-assembled with linoleic acid-modified methoxy-polyethylene glycol (mPEG-LA). miR-124 was then incorporated through electrostatic interactions with the cationic polymer MTX-PEI-LA. miR-124 could escape from endosomes and downregulate the nuclear factor of activated T cells cytoplasmic1, and the combination of miR-124 and MTX exerted superior therapeutic efficacy through anti-inflammatory and direct bone-protecting effects. Similarly, PLGA microspheres coencapsulating ketoprofen and miR-124 achieved synergistic effects in preventing inflammation and bone damage and displayed a remarkably advanced activity compared with the delivery of miR-124 or ketoprofen [107]. miRNA-21 has potent anti-inflammatory and pro-resolution properties; however, it cannot foster tissue repair and function recovery in the post-resolution stage, which is equally crucial for RA treatment [108]. The miRNA-21 + IL-4 combination showcased complementary functions in inhibiting inflammation and fostering resolution. Deng and co-workers developed inflammation-instructed nanocomplexes for co-delivering miR-21 and IL-4 in a hierarchical manner to orchestrate the osteoimmune microenvironment against RA. These nanocomplexes offered an effective strategy for the programmed delivery of drug/gene cargoes [49].

Compared with RNA, pDNA offers several advantages including easier and cheaper production, longer half-lives, and lower probability of immune system activation [89]. Furthermore, pDNA vectors can be engineered to express sequences processed in the cell to produce siRNAs, which indicates their greater flexibility. Apart from the challenges encountered by the siRNA delivery system, DNA delivery systems must achieve effective nuclear entry for efficient delivery. During inflammation, M2-type macrophages secreted anti-inflammatory factors such as IL-10, in response to M1 macrophage-produced pro-inflammatory factors [109]. Increasing IL-10 levels and decreasing pro-inflammatory factor levels may be a reassuring strategy for mitigating RA by promoting M1-to-M2 macrophage polarization. IL-10 cytokine-encoding plasmid DNA (IL-10 pDNA) is efficient in anti-inflammation by expressing IL-10. Zheng et al. therefore combined IL-10 pDNA with the chemotherapeutic drug betamethasone sodium phosphate (BSP) and co-encapsulated them using HSA, a biomimetic natural material with the ability to actively target inflamed joints [110]. The co-delivery nanoparticles significantly augmented the therapeutic effect by synergistically promoting M1-to-M2 macrophage polarization. In contrast to HSA, M2 macrophage-derived exosomes (M2 Exo) exhibit carrier capabilities and anti-inflammatory properties. Li et al. encapsulated IL-10 pDNA and BSP into the M2 Exo lipid bilayer to form M2 Exo/pDNA/BSP nanoparticles, and their anti-inflammatory and therapeutic effects were investigated both in vitro and in vivo [111]. The co-delivery nanoparticles accumulated effectively at the inflamed joints, with the synergistic action of IL-10 pDNA and BSP exerting potent therapeutic effects. This promoted M1-to-M2 macrophage polarization by reducing the secretion of pro-inflammatory cytokines (such as IL-1β and TNF-α) and increasing IL-10 cytokine expression. This work proposed an effective strategy for the engineered exosome-mediated co-delivery of pDNA and chemotherapeutics through multiple mechanisms for RA treatment. Nucleic acid aptamers comprising short single-strand DNA or RNA oligonucleotides have been developed as potent therapeutic agents for RA as they can bind target molecules with high affinity [112]. The DEK target aptamer DTA and tumor necrosis factor receptor-1 (TNFR1) target aptamer Apt1-67 were combined, equipped with sticky ends to hybridize with the ATP aptamer, and fabricated as a dual-target ATP-responsive DNA nanodrug [113]. In vivo studies have demonstrated that the DTA + Apt1-67 combination exhibited better anti-RA efficacy than monotherapy. Thus, aptamer-based DNA nanodrugs are an intelligent therapeutic strategy for RA.

To sum up, nucleic acid-based gene therapy is a novel treatment targeting specific pathological pathways or genes. It exerts targeted therapeutic effects and prevents damage to the systemic immune system. Along with being loaded into or on the surface of aforementioned nanocarriers, nucleic acids can be used to functionalize membranes or liposomes and thus induce membrane fusion for its properties in recognition and dynamic reconfiguration. This may allow selective and spatiotemporal fusion and drug delivery into cells [114]. This is a promising strategy for reprograming cells for combination therapy, and additional studies are warranted in this direction. Nucleic acid-based co-delivery systems are developed for augmented efficacy and can serve as a gene-engineered and functionalized precision therapy platform.

**Table 3:** Co-delivery with nucleic acids

Nucleic acid	Other agents	Co-delivery system	Admini-stration	Mechanisms (downregulation [↓]/upregulation [↑] )	Ref.
TNF-α siRNA	Alpha-tocopheryl succinate	Generation 5 poly(amidoamine) dendrimer-entrapped gold nanoparticle	Intra-articular	•↓TNF-α •Scavenge ROS •Ameliorate joint destruction	[92]
TNF-α siRNA	Artemisinin	Artemisinin derivatives self-assembly nanoparticles and hydrogel	Intra-articular	•↑AMPK •↓NF-κB, NLRP3, TNF-α, IL-1β	[82]
p65 siRNA	Dexametha- sone	Polymeric hybrid micelles	Intravenous	•↓NF-κB •↓TNF-α, IL-1β, iNOS •Repolarize macropha- ges from M1 to M2	[94]
Notch-1 siRNA	Methotrexate	Polymeric micelles	Intravenous	•↓Notch-1	[97]
Mcl-1 siRNA	Dexametha- sone	Folate-conjugated polymeric micelles	Intravenous	•↓TNF-α, IL-1β,	[100]
Mcl-1 siRNA	Dexametha- sone	HA-coated pH-responsive nanoparticles	Intravenous	•Reduce swelling and erythema	[101]
Mcl-1 siRNA	Tirapazamine	Mesoporous silica nanoparticles	Intravenous	•↓TNF-α, IL-1β, IL-6 •↑IL-10 •Kill activated macrophage	[102]
MMP-9 siRNA	Indomethacin + Methotrexate	Polymeric nanoparticles and hydrogel	Intravenous	•Redude swelling and erythema •↓TNF-α, IL-6, MMP-9 •Reverse cartilage disruption	[103]
MicroRNA-124	Methotrexate	Linoleic acid-modified branched PEI and PEG nanoparticles	Intravenous	•↓TNF-α, IL-1β, IL-17	[106]
MicroRNA-124	Ketoprofen	PLGA microspheres	Subcutaneous	•Relieve pian •↓RANKL	[107]
MicroRNA-21	IL-4	Core-shell nanoparticles	Intravenous	•Repolarize M1→M2 •↓ NF-κB	[49]
IL-10 pDNA	Betamethaso- ne sodium phosphate	M2-type exosomes	Intravenous	•↓TNF-α, IL-1β, •↑IL-10 •Repolarize M1→M2	[111]
Apt1-67	DTA	Self-assembly nanodrug	Intravenous	•Inhibit caspase-3 and NETs formation	[113]

## Nanotechnology-empowered combination therapy with therapeutic nanomaterials

Although multiple therapeutic agents can be co-delivered using nanocarriers to synergistically treat RA, drug-related side effects, DDIs, and premature leakage from nanocarriers may result in unsatisfactory efficacy. As nanotechnology advanced rapidly, higher requirements are proposed for the versatile functions of nanomaterials, which include but are not limited to potent drug carriers. In fact, because of their specific compositions and properties, nanomaterials may offer prospective therapeutic efficacy. This section provides an overview of integrating therapeutic agents and nanomaterials with medical effects, including anti-inflammation, oxidative stress regulation, and bone tissue repair, which may extend combination therapy to a more functional direction.

### Anti-inflammation

Chronic inflammation is a crucial player in RA progression. Although numerous anti-inflammatory drugs are available, these drugs are generally associated with adverse effects such as gastrointestinal and kidney dysfunctions [115]. Multiple nanocarriers have been fabricated to maximize the efficiency and minimize the toxicity of combined therapeutics. However, loading and delivering efficiencies are always unsatisfactory. Nanomaterials with anti-inflammatory efficacy have received increasing attention because of their potential to regulate inflammatory cells, inhibit inflammatory substances, and target inflammatory pathways.

Chrysotherapy, which involves the use of gold compounds, is a method used for RA treatment for a long time, while auranofin and sodium aurothiomalate are current commercial therapies [116, 117]. Gold nanoparticles (Au NPs) can serve as nanocarriers and exert anti-inflammatory effects. Au NPs with a 25-nm diameter represent the most effective treatment that significantly reduces inflammation, decreases bone and cartilage erosion, and prevents pannus formation [118]. Furthermore, Au NPs significantly overcame MTX-related drug resistance and enhanced its therapeutic effectiveness [119]. By conjugating MTX to Au NPs, Rafik et al. evaluated its effects on rheumatoid vascular dysfunction (RVD) [120]. They noted that the immunomodulatory action of MTX combined with the anti-atherogenic potential of Au NPs promptly controlled the whole features of RVD. To treat multiple RA symptoms by using Au NP-based nanoplatforms, Keshtiara et al. constructed a multifunctional core-shell nanostructure consisting of tragacanth, frankincense, tannic acid, 10-hydroxy-2-decanoic acid (10-HDA), and Au NPs [121]. Molecular docking showcased the interactions of 10-HDA and Au NPs with NF-*κ*B signaling pathway proteins. In an in vivo study, 10-HDA and Au NPs were confirmed to exert the synergistic anti-inflammatory effect through the NF-*κ*B signaling pathway. In addition to Au NPs, Au clusters have exhibited an anti-inflammatory impact equivalent to that of MTX. PEGylated gold nanorods can target the cytokinesis of endothelial cells, ultimately inhibiting angiogenesis [122]. Further studies are warranted to compare the anti-inflammatory effects of Au with diverse morphologies and explore the underlying mechanisms.

Silver nanoparticles (Ag NPs) are highly commercialized nanomaterials for biomedical use because they can be easily synthesized and modified, and have diverse biological activities [123]. Ag NPs have anti-inflammatory efficacy on their own [124]. Folic acid-modified Ag NPs actively targeted M1 macrophages to induce M1 macrophage apoptosis and M2 macrophage polarization, ultimately realizing effective RA treatment [125]. However, Ag NPs are usually synthesized using chemical-reducing agents, which have adverse biological effects. Synthesizing Ag NPs by using plant extracts as reducing and stabilizing agents is advantageous because of low cost, eco-friendliness, and ease of operation [126]. Guggul-mediated biosynthesized Ag NPs (G-Ag NPs) exhibited good in vivo safety when administered orally, and the anti-arthritis efficacy was comparable to that of MTX [127]. Likewise, on investigating the potential anti-arthritis effects of hesperidin loaded in gum acacia-stabilized green Ag NPs [128]. Rao et al. confirmed the potent inhibitory capacity of the drug nanoparticles by intervening in the rheumatic mechanism through the prevention of cellular infiltration into the inflamed synovium and interference with the expression of TLR-2 and TLR-4 immune receptors.

**Fig. 6:**
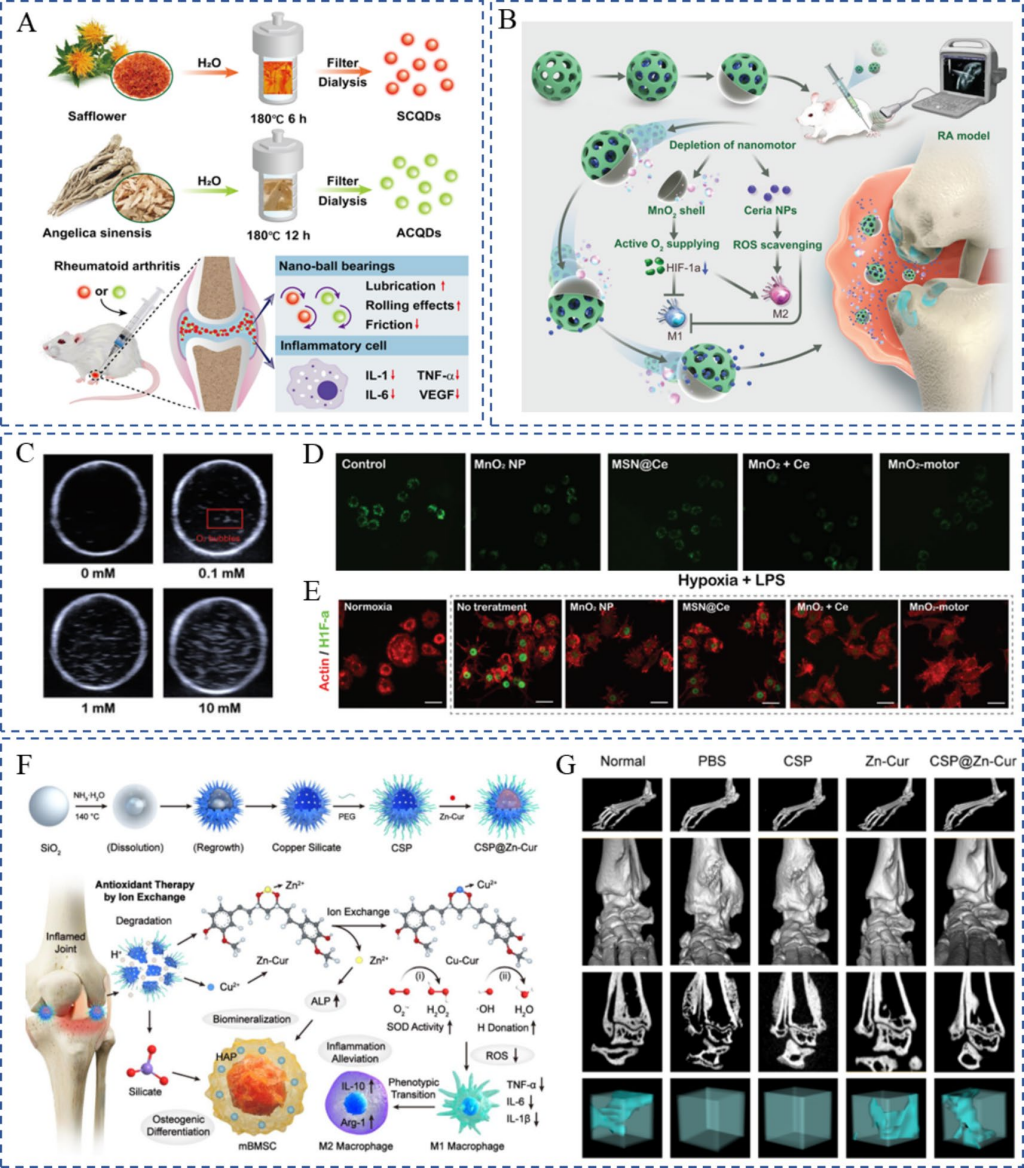
Nanoparticles with different therapeutic effects. (**A**) Schematic diagrams illustrating the fabrication of herb-derived QDs and the anti-inflammation effects in vivo. Reproduced with the permission copyright © 2023 American Chemical Society [129]. (**B**) Schematic illustration of in vivo therapeutic mechanism using MnO_2_-motors for hypoxic-inflamed joints through active O_2_ generation and ROS scavenging. (**C**) Ultrasound detection of the generated oxygen in vitro. (**D**) Inverted fluorescence microscopy images of intracellular H_2_O_2_ in RAW264.7 cells. (**E**) HIF-1𝛼 staining of RAW 264.7 cells. Reproduced with the permission copyright © 2023 American Chemical Society [130]. (**F**) Schematic illustration of the synthesis and therapeutic mechanism of CSP@Zn-Cur nanomedicine. (**G**) Micro-CT scanning and 3D reconstructed images of ankle joints of mice in different groups. Reproduced with the permission copyright © 2023 WILEY-VCH [131]

Herb-derived carbon quantum dots (CQDs), such as safflower CQDs and *Angelica sinensis* CQDs, yielded ultrahigh lubrication and anti-inflammatory efficacy [129]. These CQDs primarily consist of C, O, S, and N atoms, which are similar to those of the precursors and have inherent anti-inflammatory properties. Safflower and *A. sinensis* CQDs displayed extraordinary anti-inflammatory efficacy in relieving swelling-related symptoms and inhibiting the expression of related inflammatory cytokines such as IL-1, IL-6, and TNF-α (Fig. 6A). This attractive strategy encourages the construction of therapeutic nanomaterials by using eco-friendly “green” methods on herbs. Inspired by this, plant-derived vesicles may possess therapeutic abilities similar to those of their precursors, and the potential benefits of herbal remedies in combating arthritis must be explored.

Apart from Au and Ag, other metal nanomaterials also exert anti-inflammatory effects, although they have not yet been combined with chemotherapeutics for RA treatment. For instance, PEG-, RGD-, and Ru-modified selenium NPs can induce nitric oxide (NO) to regulate inflammatory responses and inhibit angiogenesis [132]. Moreover, of note, nanomaterials may also display pro-inflammatory activities. Typically, data regarding whether Fe_3_O_4_ NPs can exhibit pro- or anti-inflammatory activities are conflicting. According to recent studies, Fe_3_O_4_ NPs do not induce a respiratory burst by themselves, but they can alter the biology of human neutrophils and exert pro-inflammatory effects [133]. Further studies are warranted for investigating the potential undesirable effects of nanomaterials in vivo and further elucidating the mechanism underlying synergistic anti-inflammatory effects between nanomaterials and drugs.

### Regulation of oxidative stress

In RA, inflammatory cell infiltration leads to hypoxia and ROS overproduction. This causes oxidative damage to articular cells and activates macrophage polarization toward the pro-inflammatory M1 phenotype, which results in the release of various inflammatory factors, thereby aggravating synovial inflammation. Key antioxidant enzymes, such as superoxide dismutase (SOD), catalase (CAT), and glutathione peroxidase (POD), play vital roles in regulating oxidative stress at a reasonable level [134]. The use of nanomaterials for loading natural enzymes to modulate the oxidative stress microenvironment has been extensively investigated [135, 136]. For example, using liposomes, CAT was co-encapsulated with MTX, where CAT catalyzed the conversion of elevated intracellular hydrogen peroxide levels into oxygen and water. Then, continuous generation of oxygen inside the liposomes eventually disorganized its structure adeptly, and the encapsulated MTX was released to synergistically exert anti-RA effect [137]. However, natural enzymes have many intrinsic fragilities in terms of selective stability, strict physiological concentrations, and poor reusability. Nanozymes have attracted extensive attention because of their simple preparation method, low cost, and high stability, even under harsh conditions. To date, noble metals, metal oxides, and carbon-based nanoparticles with unique catalytic activities have been extensively explored for their abilities to regulate oxidative stress and remodel the RA environment.

The MnO_2_ nanozyme can mimic CAT and SOD activities, and thus reduce oxidative stress and hypoxia. Wu et al. synthesized a high-performance nanozyme of polydopamine/MnO_2_ (PDA@MnO_2_) and co-loaded it with MTX by using polymeric microneedles [138]. When inserted into the skin tissue, the microneedles degraded. The PDA@MnO_2_ served as a robust antioxidant that effectively removed ROS and reduced inflammation through synergistic effects with MTX-mediated chemotherapy. Moreover, MnO_2_ serves as a drug carrier, allowing the loading of different drugs into its hollow structure. Jia et al. successfully used MnO_2_ for loading dexamethasone sodium phosphate and collaborated to reprogram the joint site environment to near-normal physiological conditions [139]. To reduce toxicity and targeted delivered to inflamed joints, macrophage-derived microvesicles were ingeniously employed to disguise MnO_2_, thereby forming a “messenger nanozyme” that actively targeted inflammatory tissues and accumulated in activated macrophages, fibroblasts, and chondrocytes. Finally, it showed potent properties that facilitated restoring O_2_^−^ and H_2_O_2_ metabolism. The messenger nanozyme also promoted macrophage repolarization and inhibited the TNF-α and IL-1β feedback loops among inflammatory cells. In this study, a novel therapeutic approach against RA was established with nanozymes serving as both therapeutic agents and carriers, which may be universal to other inflammatory diseases. Apart from being co-delivered with drugs, nanozymes can be combined with stem cells to augment the efficacy of stem cell therapy. For instance, the mesoporous manganese cobalt oxide (MnCoO) nanozyme derived from Mn_3_ [Co(CN)_6_]_2_ metal-organic frameworks (MOFs) has been combined with bone marrow-derived mesenchymal stem cells to protect implanted cells from ROS and hypoxia-mediated death and osteogenic limitation [140]. This is a promising strategy for solving the persistent challenge of stem cell transplantation and revolutionizing conventional intervention.

Ceria oxide-based nanostructures are considered competent ROS decomposers because of their low cytotoxicity and high catalytic efficiency in eliminating superoxide. This is achieved by cycling between Ce^4+^ and Ce^3+^ in oxidation states. Zhou et al. developed a ceria oxide nanozyme-integrated rhein-loaded biopolymer-based micelle [141]. Specifically, the Ce^3+^/Ce^4+^ redox pair presented SOD-like enzymatic activity to rapidly decompose ROS and alleviate oxidative stress in M1 macrophages, and rhein inhibited TLR4 signaling in the M1 macrophages. They cooperated to induce repolarization into the anti-inflammatory M2 phenotype, thereby ameliorating local inflammation and promoting cartilage repair. However, of note, the active sites of nanozymes must be sufficiently exposed so that the enzymes exhibit catalytic activity, which might be suppressed by the complicated modification of surface engineering. Accordingly, Liu et al. intelligently developed a Janus nanoplatform comprising a CeO_2_-Pt nanozyme subunit on one side and the micheliolide-loaded periodic mesoporous organosilica subunit on the other side [142]. The Janus structure allowed the exposure of more active sites and thus enhanced the ROS scavenging capability of the CeO_2_-Pt nanozyme compared with its core-shell counterpart. Likewise, nanomotors made of ceria and the MnO_2_ half-shell were synthesized to regulate the RA microenvironment by consuming overproduced H_2_O_2_ and generating O_2_ synergistically (Fig. 6B) [130]. Nanomotors can effectively produce O_2_ and alleviate hypoxia, synovial inflammation, bone erosion, and cartilage degradation in joints (Fig. 6C–E).

Prussian blue nanoparticles (PB NPs) are effective ROS scavengers that display multi-enzyme–activity, including POD, CAT, and SOD. Additionally, these nanoparticles exhibit superior biocompatibility and higher drug-loading efficiency. Chen et al. reported the use of the siRNA + PB NPs combination to reduce TNF-α/IL-6 levels and scavenge ROS from the RA microenvironment [143]. To augment the targeting ability and biocompatibility of PB NPs, they prepared biomimetic nanoparticles by using macrophage membrane vesicles. Clinical microcomputed tomography and histological analysis confirmed the effectiveness of the aforementioned treatment. Similarly, to achieve effective RA treatment, a multifunctional nanoplatform was developed by combining schisanlactone E (an anti-RA compound isolated from Tujia ethnomedicine *xuetong*) and PB NPs, which exhibited the anti-inflammatory effect and ROS scavenging capacity [144]. A hybrid membrane based on red blood cells and fibroblast-like synoviocytes was used for coating nanoparticles to improve their immune escape and allow targeting of the arthritis joint owing to the “homing effect.” In vitro and in vivo assays have demonstrated the outstanding performance of multifunctional nanoparticles in inhibiting synovial hyperplasia and bone destruction, partly by suppressing the NF–κB signaling pathway and MMP expression.

RA involves the downregulation of antioxidases, and directly administered natural antioxidases are limited by their short half-lives and poor stability. Nanozymes are analogous to natural antioxidases for various catalytic–therapeutic purposes and can effectively treat RA by regulating oxidative stress and improving the original disorganized microenvironment. Generally, surface property-related factors, such as morphology, size, composition, surface lattice, and modification, determine the enzymatic properties of nanozymes. Furthermore, increased efforts should be made to understand the reaction mechanisms and rational design of bio-inspired nanozymes, while exploring their new biological and biomimetic applications in combination therapy for RA.

### Bone tissue repair

Anti-inflammatory treatment is often a priority for conventional pharmaceutical therapies, and joint destruction may be neglected. Nano-based bone tissue engineering serves as a strategy with great potential for reducing bone erosion in RA progression [145, 146]. Nanomaterials with components similar to bone or biomineralization properties have been extensively investigated.

Nano-sized hydroxyapatite (HAP) exhibits characteristics similar to those of bone constituents and is a source of calcium phosphates required for repairing bones damaged during the disease course. Hyaluronate-functionalized HAP nanoparticles were used to co-deliver teriflunomide and MTX. The drug-carrying nanoparticles demonstrated considerable efficacy in preventing disease progression and promoting articular regeneration [72]. In addition to the directly used HAP, components released from the nanoplatform formed HAP by providing PO_4_^3−^ and Ca^2+^ to facilitate mineralization. A core-shell nanostructure was developed by incorporating copper silicate nanoparticles and Zn-curcumin, which served as the shell and inner core, respectively (Fig. 6F) [131]. The silicate + Zn^2+^ combination was advantageous in promoting osteogenic mineralization, because silicate promoted Ca^2+^ absorption, while Zn^2+^ elevated PO_4_^3−^ production, and cooperated to promote osteoblast biomineralization (Fig. 6E). This ion-exchange strategy augmented the anti-arthritis effect of Cur and facilitated bone tissue repair.

Biphasic calcium phosphate (BCP), a combination of HAP and β-tricalcium phosphate, also exhibits bone differentiation and preferential biodegradation properties. After designing a salic acid (SA)-modified chitosan oligosaccharide-based BCP, Xu et al. then loaded MTX into this system to treat RA through tissue engineering [147]. Chitosan oligosaccharides exert chondroprotective effects. SA and BCP synergistically mineralized calcium phosphate, thereby allowing more osteoblasts to adhere, proliferate, and differentiate. After the drugs were intra-articularly administered, BCP underwent degradation in the acid microenvironment of arthritic joints, and MTX was immediately released to remarkably suppress the inflammatory response. This well-designed MTX-loaded BCP nanoplatform synergistically reduced inflammation and repaired bone tissues, thereby serving as a novel strategy for RA treatment via nanometer-scale dimensions mimicking natural tissues.

Black phosphorus (BP) nanomaterials have attracted considerable attention because of their potential osteogenic properties and their degradation products are bone tissue components and benefit mineralization [148]. Moreover, BP nanomaterials have good biocompatibility and are biodegradable in the physiological environment [149]. A novel therapeutic thermo-responsive hydrogel co-loaded with BP nanosheets (BPNs) and platelet-rich plasma (PRP) was developed for RA treatment [150]. BPN degradation accelerated PO_4_^3−^ release and subsequently promoted new calcium phosphate formation to facilitate biomineralization performance. In an in vivo study, the BPN/chitosan hydrogel markedly reduced the cartilage and bone destruction level, thereby offering a new strategy for RA treatment through phosphorus-driven osteogenesis.

Zeolites have also garnered considerable interest as a novel therapeutic option against bone abnormalities including RA. They have been commonly accepted as medical devices in various clinical settings. The combined use of zeolite nanoparticles and vitamin B12 significantly improved the histopathological implications of articular joints of complete Freund’s adjuvant-induced arthritic rats [151]. Furthermore, zeolites can exert anti-arthritis, anti-inflammatory, and antioxidant effects, possibly leading to more encouraging outcomes when used in combination therapy.

Progressive deterioration of joint structures occurs gradually over a period of weeks to months and results in deformity, functional incapacity, and disability. Current treatment strategies primarily focus on existing inflammation, and few drugs are optional for bone repair. Combining drugs and nanomaterials with bone tissue repair properties is markedly promising in delaying RA progression through both anti-inflammation and bone regeneration.

### Nanotechnology-empowered multimodal therapy

Pharmaceutical therapy has evolved from monotherapy to combination therapy in RA treatment. However, chemotherapy-based single-mode therapy remains insufficient in addressing RA intractability and is inevitably associated with drug-related side effects. Advanced nanotechnology has led to the rapid development of multimodal systems, where different imaging and therapy modalities are integrated into a single nanoplatform, yielding multiple functions. Early RA diagnosis and monitoring the treatment efficiency are possible because of the combining of imaging techniques, such as fluorescence imaging (FI), photoacoustic imaging (PAI), and magnetic resonance imaging (MRI). Integrating different therapy modalities, such as chemotherapy, phototherapy, SDT, and microwave therapy, complemented by maximizing the advantages of each mode and offering ostentatious superadditive therapeutic effects. This section discusses in detail the combination of different imaging or/and therapy modalities, emphasizing the construction of multifunctional nanoplatforms for multimodal therapy and the exploration of the underlying mechanism.

### Theranostics

Early RA diagnosis is critical to avoid further complications and reversible disability in patients. Nowadays, researchers are no longer working toward therapy alone, they are focusing on emerging theranostics aiming to bridge the gap between diagnosis and treatment [152]. Imaging techniques with higher spatiotemporal resolution and signal-to-noise ratio are highly desirable to precisely diagnose RA. Nanotechnology has many advantageous properties, such as tunable optical, electrical, and magnetic properties for diagnosis [153]. These properties render early diagnosis and real-time feedback of actual treatment effects possible. Extensive efforts have been taken to construct nano-based high-performance theranostic platforms to diagnose RA early and treat it effectively.

MRI is a commonly used clinical imaging modality and is termed as the “gold standard”. Normal and abnormal tissues can be differentiated with MRI because of the use of contrast agents. However, conventional MRI usually lacks sufficient sensitivities and accuracies for detecting RA at an early stage. Superparamagnetic iron oxide nanoparticles (SPIONs) are versatile MRI contrast agents. To achieve targeted therapy and diagnosis, MTX and SPIONs were co-encapsulated using CD64 monoclonal antibody-functionalized solid lipid nanoparticles. These nanoparticles exhibited marked promise in future theranostic applications [154]. Instead of delivering SPIONs using targeted nanoparticles, Zhang et al modified SPIONs with the peptide VHPKQHR, which exhibited a high affinity to vascular cell adhesion molecule-1 (VCAM-1) [155]. In vivo MRI and biodistribution results revealed that the synthesized contrast agents effectively targeted VCAM-1 for early-stage RA diagnosis and generated high contrast on T1-weighted MRI.

**Fig. 7:**
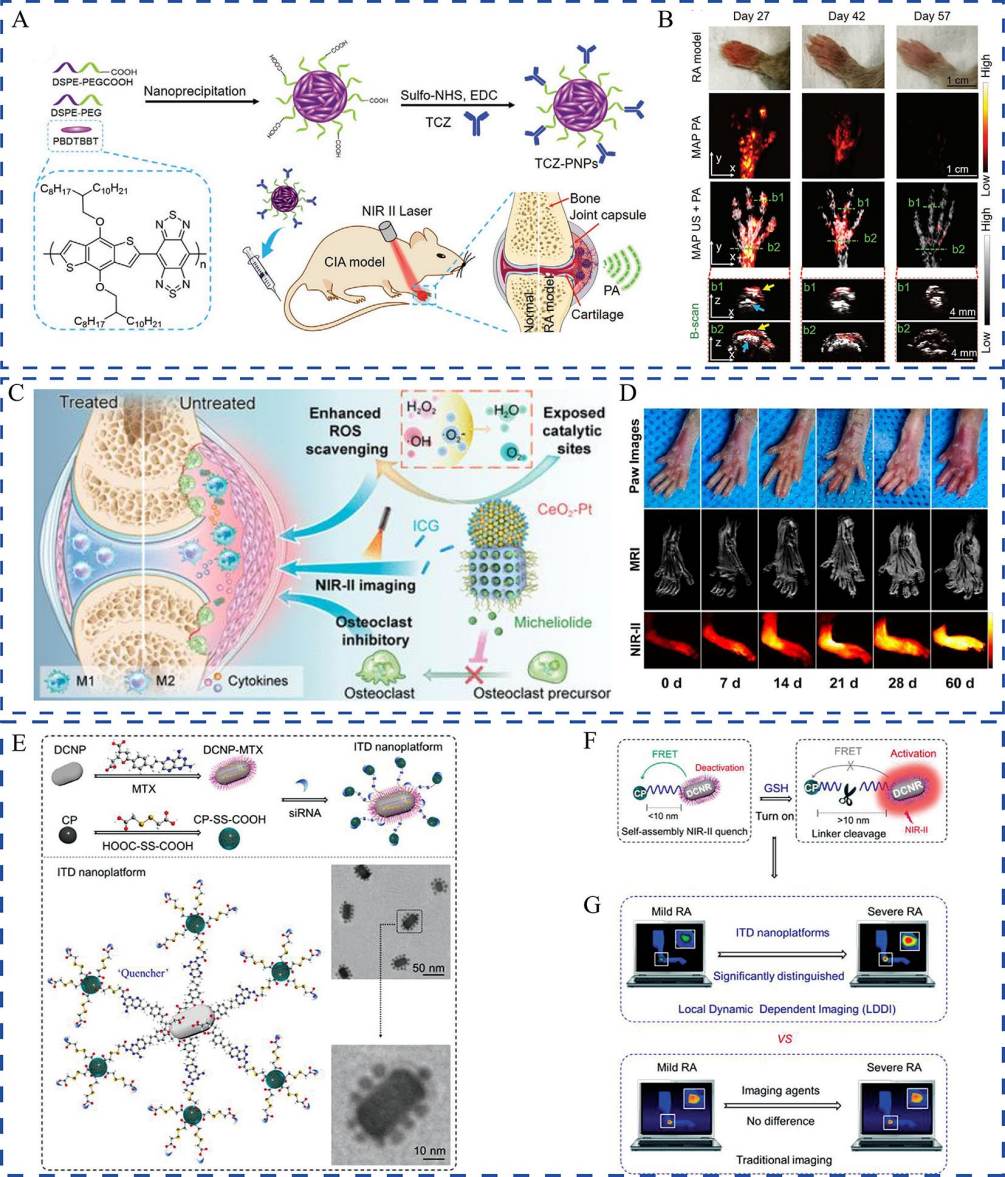
Nanotechnology-empowered combination therapy to integrate treatment and diagnosis. (**A**) Schematic illustration of TCZ-PNPs for PA imaging. (**B**) Photos and PA images of forepaws in TCZ-PNPs treated RA model groups. The B-scan PA images correspond to the green dashed region. The yellow arrow indicates swollen tissues and the blue arrow indicates cartilage tissues. Reproduced with the permission copyright © 2020 WILEY-VCH [157]. (**C**) Schematic diagram of the synthesis of Janus nanoplatform and its capability of early diagnosis and synergistic treatment of RA. (**D**) Photos, MR, and NIR-II images of the hind paws. Reproduced with the permission copyright © 2023 American Chemical Society [142]. (**E**–**F**) Illustration of the light-up process of FRET effect between CPs (serves as a “quencher” in NIR-II imaging) and DCNR. (**G**) Comparison of LDDI and conventional imaging mode on the RA model. Reproduced with the permission copyright © 2022 John Wiley & Sons [158]

Near-infrared-II (NIR-II, 1000–1700 nm) photoacoustic (PA) molecular imaging is considered a reassuring strategy because of its high sensitivity and specificity at large penetration depth, which allows effective diagnosis and treatment guidance without radiation risk [156]. The PA signal is generated when light is absorbed by a molecule under short-pulse laser irradiation, which overcomes the common optical diffusion limit. To achieve PA-imaging guided RA therapy, new NIR-II-conjugated polymer nanoparticles (PNPs) with strong light absorptivity, high photostability, and good biocompatibility were synthesized. The monoclonal antibody tocilizumab (TCZ), which has RA therapeutic and targeting functions, was conjugated to PNPs (TCZ-PNPs) to integrate RA-targeted concurrent imaging and therapy (Fig. 7A) [157]. TCZ-PNPs exhibited excellent targeting ability and allowed the effective noninvasive diagnosis of arthritis joints with a high signal-to-noise ratio of 35.8 dB in three-dimensional (3D) PA tomography images (Fig. 7B). After 1-month treatment and monitoring of TCZ-PNPs, RA was significantly suppressed, while the therapeutic evaluation was consistent with clinical micro-CT and histological analyses.

NIR-II FI is a potent imaging modality for RA diagnosis because it is real-time and offers superior spatial resolution, considerable depth, and dynamic tracking of changes in interest regions [159]. Biocompatible indocyanine green (ICG) has been extensively used for NIR-II FI in clinical practice because of its tailing emission in the NIR-II window. Jin et al. proposed an innovative NIR-II FI-guided safe-dose chemoradiotherapy strategy for developing a multimodal theranostic platform for RA [160]. MMP-9-responsive nanoradiosentizer-40 was designed by encapsulating cisplatin, an ICG, in a gelatin shell, which facilitated responsive “turn on” NIR-II fluorescence in the unique microenvironment of arthritis joints. The released ICG illuminated the accurate target volume delineation for guiding radiotherapy and served as a real-time reporter quantifying cisplatin release, thereby allowing efficacy monitoring. Cisplatin functions as an effective chemotherapeutic agent and a radiosensitizer for RA through the dual ROS-mediated mitochondrial apoptotic pathway. This activatable NIR-II FI-guided chemoradiotherapy strategy provided a novel paradigm for precise RA theranostics, which may be adapted to other refractory benign diseases. Likewise, a Janus nanoplatform comprising the CeO_2_-Pt nanozyme, micheliolide, and ICG was developed for the synergistic therapy and early diagnosis of RA (Fig. 7C) [142]. By taking advantage of NIR-II FI of ICG, the Janus nanoplatform displayed desired effectiveness in detecting RA lesions as early as 14 days, whereas the “gold standard” MRI showed no visible signals until 28 days (Fig. 7D).

However, a single imaging modality is sometimes insufficient for obtaining integrated information. In such cases, multimodal imaging techniques are proposed, which involve taking advantage of several modalities to image the same or different targets. To target inflamed joints, evaluate disease progression, and monitor therapeutic efficacy simultaneously, He et al. fabricated a novel PLGA nanoplatform co-encapsulating the ultrasound contrast agent perfluoropropane and the fluorescent and photoacoustic contrast agent ICG, along with MTX [161]. The platform was effective in providing appropriate guidance and monitoring for subsequent treatment under ultrasound and PA imaging following laser- and low-intensity-focused ultrasound excitation. This is thus a feasible and promising approach for multimodal imaging navigation and efficient RA treatment. Considering that the complicated pathological microenvironments of RA are interfering, advanced diagnosis with improved accuracy and the distinguishability of the disease status needs to be developed further. Li et al. established an inflammation-triggered disassembly (ITD) of the nanoplatform accompanied by local dynamic-dependent imaging (LDDI) to efficiently and cooperatively treat RA [158]. As a local dynamic response to specific inflammatory milieus, endogenous glutathione (GSH) breaks the S–S bond to ensure light-up activation of the ITD nanoplatform. LDDI was performed on the basis of the GSH-mediated fluorescence resonance energy transfer (FRET) effect between core-shell compositions (Fig. 7E–G). This study demonstrated the potential of the inflammation-triggered disassembly strategy to treat RA effectively and achieve a local dynamic-dependent diagnosis.

### Phototherapy

RA synovial tissues show excessive cell proliferation, strong metabolism, and aggressiveness. Phototherapy, including photothermal therapy (PTT) and photodynamic therapy (PDT), induces the apoptosis or necrosis of inflammatory cells infiltrating the arthritis cavity by generating heat or ROS, thus reducing inflammatory factors, synovial erosion, and joint destruction [162]. Phototherapy is an effective and safe modality because light irradiation can be spatially and temporally controlled to noninvasively ablate the hypertrophic synovium. Moreover, nanomaterials with photothermal or photocatalysis characteristics have broad development prospects.

In PTT, photothermal transduction agents are used to absorb light of a specific wavelength while the absorbed energy is converted into heat through nonradiative attenuation [163]. PTT can increase the temperature of the surrounding environment and irreversibly damage disease-related cells, such as activated macrophages and fibroblasts. Chen et al. fabricated a core-shell nanocomposite to effectively treat RA by combining PTT and chemotherapy [164]. Quadrilateral ruthenium nanoparticles (QRu NPs) were prepared as the core. Serving as the shell, thermosensitive PLGA vesicles simultaneously encapsulated resveratrol through hydrophobic interactions. The QRu NPs demonstrated excellent photothermal properties, which inhibited M1 macrophage’s recruitment and infiltration ability and resulted in thermosensitive release of resveratrol at the inflammation site to promote M2 macrophage polarization. Similarly, Xu et al. synergistically treated RA through a combination of hexagonal palladium nanosheet-based PTT and MTX-based chemotherapy [165]. According to an in vivo study, PTT offered a compensatory effect to MTX and ultimately exerted compelling therapeutic effects in inhibiting inflammatory cytokines (TNF-α, COX-2) and VEGF-induced inflammatory response.

**Fig. 8:**
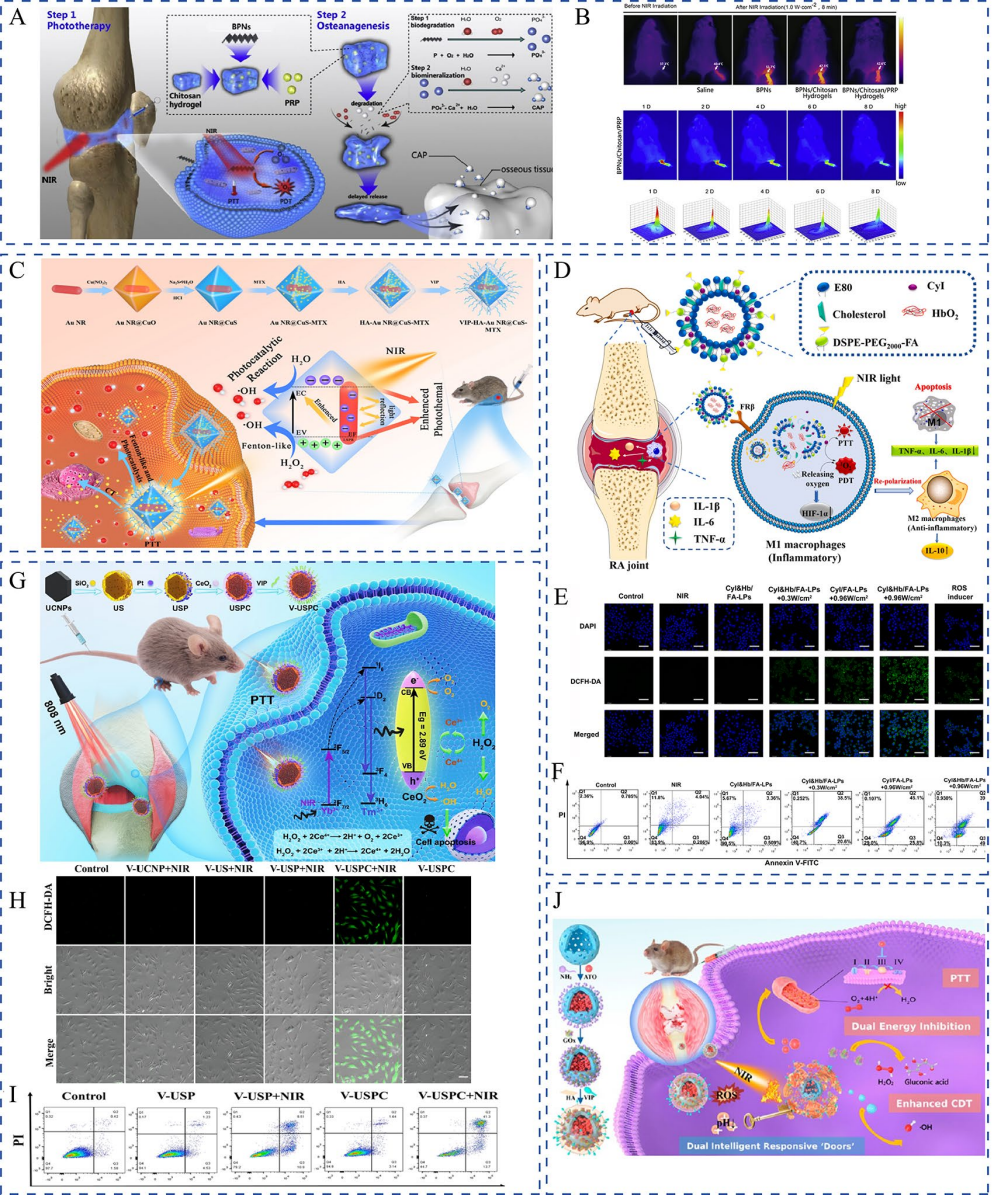
Nanotechnology-empowered combination therapy with phototherapy. (**A**) Schematic illustration of PRP-Chitosan and BPNs-based biotherapy and phototherapy. (**B**) In vivo representative photothermal images of arthritis sites under NIR irradiation. Reproduced with the permission copyright © 2020 Elsevier [150]. (**C**) Schematic illustration of MTX-based chemotherapy and Fenton-like reaction enhanced PDT/PTT. Reproduced with the permission copyright © 2021 Elsevier [168]. (**D**) Schematic diagram of synergistic phototherapy and repolarization of M1-to-M2 macrophages by oxygen supplementation liposomes. (**E**–**F**) Evaluation of ROS generation property in M1-type macrophages and their resulting apoptosis/necrosis. Reproduced with the permission copyright © 2023 Elsevier [169]. (**G**) Intelligent nano-platform that combines CeO_2_in situ oxygen production with PDT through UCNPs serve as energy donors. (**H**–**I**) Evaluation of ROS-generation property in FLS cells and their resulting apoptosis/necrosis. Reproduced with the permission copyright © 2021 Elsevier [170]. (**J**) Schematic diagram of synergistic O_2_-economized dual energy inhibition/enhanced CDT/PTT therapeutic mechanism. Reproduced with the permission copyright © 2022 American Chemical Society [136]

Unlike PTT, PDT uses a photosensitizer to convert surrounding oxygen to generate ROS with cytotoxic effects, thereby removing synovial hyperplasia [166]. PTT can enhance PDT’s efficacy, because mild hyperthermia accelerates the blood flow to augment the vascular saturated O_2_ concentration, thereby elevating the ^1^O_2_ yield in the oxygen-dependent type II PDT. A novel therapeutic platform consisting of BPNs and PRP-chitosan thermo-responsive hydrogel was developed to synergistically treat RA (Fig. 8A) [150]. Notably, BPNs exhibited illustrious PTT and PDT properties simultaneously (Fig. 8B), and the osteanagenesis potential. Meanwhile, the released PRP contained growth factors and cytokines and thus enhanced cell adhesion and proliferation. This successful combination of phototherapy and biotherapy offered a new avenue for RA treatment by excising proliferating synoviocytes and reducing bone destruction. Zhao et al. synthesized macrophage-derived hybrid exosome-mimic nanovesicles laden with black phosphorus. This system achieved comprehensive suppression of inflammation through PTT/PDT and exerted exosome-mimic nanovesicle-directed anti-inflammatory effects [167]. To augment the efficiency of PTT/PDT, Huang and co-workers simultaneously used two photothermal agents and exerted strong multifunctionality. Au nanorod (Au NR) and copper sulfide (CuS) were integrated into yolk-shell octahedral nanoparticles, followed by the loading of MTX [168]. The copper ions from CuS interact with H_2_O_2_ to produce •OH in the Fenton-like reaction, which exerts the chemodynamic effect in the CuS shell and the PDT effect by coupling CuS and Au NR (Fig. 8C). The resulting Au NR@CuS-MTX nanoparticles synergistically inhibited synovial cells and alleviated edema degree through PTT/PDT/chemotherapy.

However, because of the anoxic environment of RA synovial tissues and the oxygen required for PDT, PDT’s efficiency was always limited [171]. The RA anoxic environment is generally formed because of the imbalance between O_2_ consumption and supply. The first strategy is to use nanocarriers to directly transport exogenous O_2_ to the arthritis site. Hemoglobin (Hb) is the main red blood cell component with an inherent reversible oxygen-binding capacity. By developing M1-targeted liposomes, Zhang et al. simultaneously delivered Hb and the phototherapeutic agent (CyI) for enhanced PTT/PDT [169]. Under NIR irradiation, CyI transitions to singlet electronic states that are decayed by ^1^O_2_ and photothermal pathways to impose PDT and PTT effects (Fig. 8D). Meanwhile, Hb supplies oxygen to relieve hypoxia and subsequently enhances PDT efficiency. This study offered a system that can trigger M1 macrophage death and induce the M1-to-M2 phenotype transition in surviving macrophages (Fig. 8E, F). Nevertheless, the exogenous O_2_ delivery pathway may augment the toxic effect of ROS on normal tissues. The second strategy is to actively increase O_2_ production in situ. Nanozymes with the ability to decompose endogenous hydrogen peroxide to form O_2_ are promising candidates. Advanced upconversion nanoparticles@SiO_2_@Pt@CeO_2_ were fabricated for in situ O_2_ production/PTT/PDT treatment of RA (Fig. 8G) [170]. The combination of Pt-mediated PTT and CeO_2_-enhanced PDT inhibited the proliferation of fibroblast-like synovial cells (Fig. 8H, I). Additionally, CeO_2_ catalyzed high H_2_O_2_ levels to produce O_2_ in situ, thereby alleviating hypoxia and resisting angiogenesis. This thus offered a new method of RA treatment through the triad of O_2_/PTT/PDT. Considering that the aforementioned two strategies may fail to continuously provide sufficient amounts of oxygen for tissue consumption, decreasing O_2_ consumption in vivo is reasonable. In a proposed efficient long-acting O_2_-economized strategy, intracellular energy metabolism of FLS was inhibited. By designing a hollow mesoporous CuS NP-based smart glucose oxidase (GOx)/atovaquone (ATO) co-delivery system, Qiu et al. realized an O_2_-economized dual-energy inhibition strategy (Fig. 8J) [136]. Under NIR stimulation in an acid environment at the targeted arthritis joints, the dual intelligent responsive “doors” orderly opened to realize controllable GOx and ATO release. GOx-based starvation therapy could limit FLS proliferation by cutting off nutrient sources and competitively utilizing intracellular glucose and O_2_ levels. ATO can inhibit the mitochondrial oxidative phosphorylation system aerobic metabolism pathway to inhibit cell activity while economizing O_2_ by inhibiting cellular respiration to promote starvation therapy. Moreover, the efficacy of nanoplatforms would remarkably improve with the addition of CuS NP-stimulated chemodynamic therapy (CDT) and PTT. Finally, starvation therapy-induced upregulation of H_2_O_2_ and acid levels would promote the Fenton-like reaction of CuS NPs under O_2_-economized dual energy inhibition, which could also augment PTT and CDT efficacies.

Although phototherapy offers the advantages of adjusting the irradiation site, power, and duration, limitations remain. The PTT scope and efficacy are limited by the laser’s penetration depth. Nanomaterials with higher photothermal conversion efficiency, deeper penetration, and more allowable exposure are desired. Additionally, the PDT efficiency is limited by the anoxic environment of arthritis joints. Oxygen must be produced or economized to maximize the efficacy. Moreover, PTT and PDT induce proinflammatory events in cancer treatment. Whether phototherapy causes inflammation in RA treatment needs to be explored further.

### Sonodynamic therapy

**Fig. 9:**
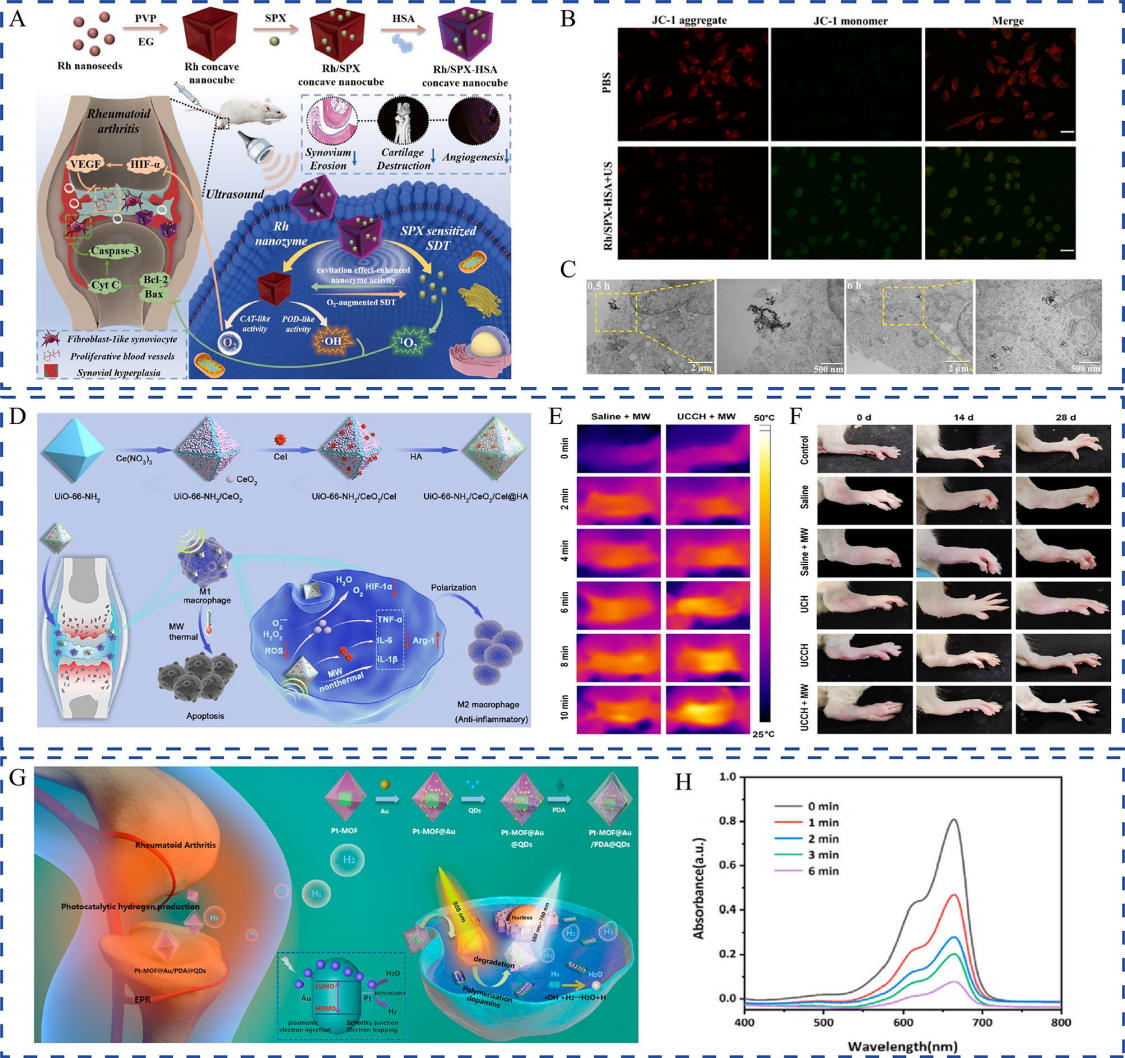
Nanotechnology-empowered combination therapy with other therapy modalities. (**A**) Schematic illustration of the preparation of Rh-SPX/HSA and its related mechanisms for the treatment of rheumatoid arthritis. (**B**) Rh/SPX-HSA induces mitochondrial membrane potential loss in FLS. (**C**) Bio-TEM images of RA-FLSs after incubation with Rh-SPX/HSA. Reproduced with the permission copyright © 2021 Elsevier [170]. (**D**) Schematic illustration of the synthetic UCCH composites and their application in ROS scavenging, O_2_ production, chemotherapy, and mild microwave thermal therapy of RA. (**E**) Infrared thermal images at the ankle site of the AIA rats. (**F**) Representative photographs of joints and claws of AIA rats. Reproduced with the permission copyright © 2023 American Chemical Society [173]. (**G**) Schematic illustration of the synthesis route and the hydrogen-photothermal treatment therapeutic mechanism. (**H**) Photocatalytic hydrogenation increased with prolonged irradiation time. Reproduced with the permission copyright © 2022 Elsevier [174]

SDT is a relatively safe and valid treatment method in which ultrasound-activated sonosensitizers are used to trigger ROS production [172]. To achieve mutual-reinforcing SDT during ultrasonic activation, Li et al. developed a concave-cubic rhodium (Rh) nanozyme doped with a sonosensitizer spafloxacin (SPX) and loaded using HSA (Fig. 9A) [170]. Rh nanoparticles are responsible for generating radicals and alleviating hypoxia to kill synovial fibroblasts as well as induce anti-angiogenesis for RA treatment. SPX causes mitochondrial dysfunction by triggering excessive ROS production, thereby suppressing fibroblasts under ultrasound conditions (Fig. 9B-C). This nanoplatform composed of SDT and the nanozyme improved hypoxia of the joint to resist angiogenesis and enormously elevated the SDT efficacy by increasing ^1^O_2_ levels. The Rh nanozyme combined with SDT therapy is promising in inhibiting fibroblast proliferation by inducing mitochondrial dysfunction. Thus, this combination of SDT and nanozyme exerts a significant curative effect and offers biological safety in RA treatment.

### Microwave therapy

Microwave therapy can improve hemolymph circulation, local oxygen supply, absorption, and dissipation of pathological metabolites, while rapidly reducing tissue swelling [175]. In microwave-induced thermal therapy, polar substances facilitate the conversion of electromagnetic energy (microwave irradiation) to thermal energy (bond vibration and rapid collision). Microwave-induced thermal therapy offers indispensable advantages compared with other hyperthermia therapies, such as deep penetration and less free radical generation [176, 177]. To inhibit M1 proinflammatory macrophages and repolarize them into M2 anti-inflammatory cells by alleviating hypoxia and scavenging ROS, Zhang et al. offered a prosing strategy based on microwave-enhanced thermal, antioxidative, and chemotherapeutic treatments. The researchers constructed a microwave-sensitive MOF of UiO-66-NH_2_ to coat CeO_2_, load celastrol, and then wrapped them with HA to form a multifunctional nanoplatform (Fig. 9D) [173]. The thermal effect of UiO-66-NH_2_-produced microwaves eliminated the excessive proliferation of inflammatory cells (Fig. 9E). By contrast, the non-thermal effect of the microwaves synergistically promoted M1 macrophage repolarization into the M2 phenotype. Meanwhile, CeO_2_ displayed SOD-like and CAT-like activities in scavenging ROS and producing O_2_, which was boosted by microwave irradiation. Moreover, microwave activation induced celastrol release for chemotherapy. According to an in vivo study, the potent effects of microwave-induced thermal therapy on relieving joint swelling and redness (Fig. 9F), as well as achieving cartilage recovery. This microwave-driven multifunctional platform showcased great potential in RA treatment, especially as a combined therapy.

### Gas therapy

Hydrogen (H_2_) is considerably advantageous in oxidative stress prevention and treatment, as it can rapidly diffuse across membranes and reduce the production of harmful ROS (especially hydroxyl radicals) in cells [178]. Therefore, H_2_ therapy neutralizes cytotoxic ROS without affecting the biological activities of other ROS. The proposed hydrogen–thermal therapy involves a combination of H_2_ therapy and PTT. An intelligent H_2_ nanogenerator based on the polydopamine- and Perovskite quantum dot (QD)-loaded MOF was constructed for the actualization of hydrogen-thermal therapy [174]. Polydopamine with excellent photothermal conversion efficiencies is used for PTT, and perovskite QDs with unique photophysical properties are used as fluorescent signals for positioning Pt-MOF@Au@QDs/PDA nanoparticles. Moreover, when the Pt-MOF@Au@QDs/PDA catalyst combines Au’s surface plasmon resonance excitation with the Pt-MOF Schottky junction, extremely efficient photocatalytic H_2_ production was observed under visible light irradiation (Fig. 9G). By combining PTT and H_2_ therapy, the PtMOF@Au@QDs/PDA effectively relieved oxidative stress in RA, significantly improved joint damage, and inhibited the overall arthritis severity.

Carbon monoxide (CO) and NO are endogenous gaseous signaling molecules with critical regulatory roles in numerous physiological processes, such as apoptosis, immune response, and neurotransmission [179, 180]. NO overexpression is a hallmark of RA, whereas CO exerts potential anti-inflammatory effects. Intriguingly, CO deactivates inducible NO synthase (iNOS) by binding to the iron center of the heme, and thus selectively depletes NO [181]. Accordingly, Tao et al. fabricated a “breathing” micellar platform that can inhale NO and exhale CO intelligently [182]. Breathing micellar nanoparticles had NO-reactive *o*phenylenediamine (*o*PDA) and CO-releasing 3-hydroxyflavone (3-HF) derivatives within the cores. These components could simultaneously deplete NO by forming benzotriazole moieties and release CO under visible light irradiation. The breathing micelles could simultaneously scavenge overproduced NO and attenuate proinflammatory cytokine levels in activated macrophages. In vivo studies have revealed that breathing micelles are more efficacious than dexamethasone in RA treatment.

## Challenges and limitations for nanotechnology-empowered combination therapy

Despite the advancements in nanotechnology-enabled combination therapy, several challenges remain. These challenges have arisen from the nanotechnology aspects and are also based on the characteristics of RA. For example, the intricacy, diversity and chronicity of RA put forward a high demand for appropriate combination candidates, treatment sequence, as well as the long-term administration safety. Candidates with varying hydrophilicity, pharmacokinetic profiles and membrane transport properties require more elaborate design in nanotechnology aspects. In this section, we discuss the challenges and limitations in detail from safety, drug-drug interactions and clinical translation aspects.

### Safety

Although extensive studies on nanoformulation-based combination therapy have been conducted, substantial concerns regarding the safety and regulatory aspects remain. Safety issues must be strictly analyzed. This is the first step for establishing a successful therapy platform from the discovery phase to its entry into clinical trials.

The first concern is the safety of nanomaterials. Nanotoxicology exists and has received considerable attention. At the cellular level, nanoparticles can cause inflammation, oxidative stress, and mitochondrial dysfunction [183]. Materials used for drug delivery are usually considered non-toxic or exhibit low toxicity. However, when transformed at a nanoscale level, the specific physicochemical properties of these materials such as size, shape, composition, mechanical properties, surface charge, and the compatibility between all materials and therapeutics potentially induce biological effects [184]. An appropriate strategy is required for evaluating the toxicity related to the interaction between nanomaterials and the biological system. How nanomaterials interact with the endothelial cell lining when administered intravenously and the arthritis microenvironment when they arrive should be unveiled. Furthermore, the safety of nanomaterials should not only be tested at the end, it is also important to foresee the risks throughout the process.

In addition, the rationality of drug combinations substantially affects the safety. A deeper understanding of the pathogenesis of RA is necessary, including the network between immune cells, non-immune cells, cytokines, chemokines and abnormal signal transduction. This will provide us the direction of combination therapy, where all components work together with the same goal to ensure the safety and efficacy. Accordingly, drug combinations with clear division of functions are selected. Adhering to the principle of clinical practice in RA is imperative. Prioritizing therapeutic agents with clinical evidence as the foundational choice is advisable. Although some combination therapy strategies have benefitted arthritis remission; in theory, the efficacy of combination therapy may be less (antagonistic), equal (additive), or more (synergistic) [185]. In another word, not all combination strategies benefit the treatment and may produce additional side effects. For example, hepatotoxicity increases when MTX is combined with long-term glucocorticoids. CYP3A4 is the key drug-metabolizing enzyme used in the treatment of inflammatory joint diseases. The combination with CYP3A4 inhibitors may increase plasma levels of the coupled drug, thus consequently increasing the risk of side effects [186]. Hence, the specific couple of drugs, different action mechanisms, ratio, drug interference, and the duration of use should all be considered.

### Drug–drug interaction considerations

According to material chemistry, hybridization of different components impairs their corresponding properties or functions, especially in a single nanocarrier. The DDI remains a major challenge when designing co-delivery systems. This highlights the need for rationale combination regimens and a precise combination ratio over the properties of multiple therapeutics. It also affects pharmacodynamics and pharmacokinetics.

In in vitro DDI, tailoring co-delivery nanoplatforms is difficult because of discrepant physicochemical properties (such as solubility, molecular sizes, polarity, and membrane transport properties) of the coupled drugs. This might also result in suboptimal pharmaceutical activity. Owing to the differences in the solubility of co-delivered payloads, nanoparticles must have an amphiphilic property or different hydrophilic/hydrophobic compositions, such as liposomes, hybrid nanoparticles, and nanocomposite hydrogels/microneedles. Molecular sizes and polarity predominantly affect the encapsulation and drug loading efficiencies, which further affect the dosage and synergistic effect. Moreover, determining the location, sequence, and release rate of different therapeutics is essential for ensuring the in vivo efficacy. The interaction mechanism between the targeted cell and the delivery system must be completely understood. In vivo DDI can affect the pharmacokinetics (absorption, distribution, metabolism, and excretion) of individual drugs. Conventional administration of drug combinations often suffers from distinct pharmacokinetic profiles of partner therapeutics, which causes an inconsistency in in vivo pharmacokinetics and biodistribution and therefore an inefficient therapy. Although co-delivery relies on the concept of combination therapy, slight differences exist between each other. For example, in clinical practice, the drugs for combination therapy are rarely administered by mixing them in the same infusion bottle, whereas in co-delivery systems, the loaded drugs enter the blood circulation at the same time. This fact poses a challenge to pharmaceutical characteristics. Although nanocarriers can transport multiple chemotherapeutics to intracellular destinations, differentiating different active targets of each chemotherapeutics remains difficult.

‘‘Jun, Chen, Zuo, and Shi” is a widely used compatibility principle for designing a traditional Chinese medicine (TCM) formula, in which one represents the main component, and the others serve as adjuvant components for increasing efficiency, reducing toxicity, or promoting the delivery of the main component [187]. Likewise, a co-delivery system can be designed according to TCM rules, where all components work together with a clear division of function. Nowadays numerous pharmacological study-based computer programs are available for assessing DDIs, which may offer useful information for designing combination regimens.

To sum up, DDI is a vital factor that should be considered when designing co-delivery systems. It affects the successful preparation of co-delivery systems with rational combination regimens, optimized ratio, and well-controlled release manner. It also influences the pharmacodynamics and pharmacokinetics of individual drugs.

### Clinical translation

Regarding the demands of treating RA synergistically, numerous nano-based combination strategies are designed and investigated in experimental RA models. However, reports on clinical settings are few, and many challenges continue to hinder its clinical translation.

The ultimate goal of nano-based combination platforms is to develop effective and safe medical products that benefit RA patients in clinics. The first concern is safety, especially for invasive administration. As mentioned above, nanocarriers and combination candidates are key factors influencing safety. A thorough, meticulous, and extended evaluation of local and systemic toxicities and immunogenicity is required to ensure the safety of nanomaterials. The second stumbling block in the translation is that nanoformulations behave unpredictably in the complex microenvironment of RA. It is difficult to ensure that multiple drugs reach the targeting site in a well-designed manner, which further hampers precise control and assessment. Furthermore, nano-based combination platforms with a fixed composition and ratio may not be able to meet different treatment demands. Differences in the study design, dosing regimens, and data from defined and specific patient populations are challenges for clinicians who treat patients with diverse characteristics and needs. In this scenario, intelligent and flexible nano-based combination platforms must be developed to release payloads responsively and precisely. Third, reaching the market is difficult for nanoplatforms because of the complicated synthesis process and the challenges associated with scaling up production. Many factors from the manufacturing device to the repeatability and stability of the product should be considered. 3D printing tools and microfluidic devices are the most extensively used strategies for improving industrial-scale manufacturing, which can be further applied to nano-based combination therapy. At last, pharmaceutical industries also pose a potential barrier, because they hesitate to cooperate in clinical trials and markets because of finance- and insurance-related concerns. On one hand, combination therapy requires special care and consideration in terms of the combination ratio, dosage, drug interaction, and additional side effects. This process involves several uncontrollable factors and the risk of failure. On the other hand, proving the superiority of nanoformulation to the bare form of combination therapy is time-consuming. A robust quality management system is generally required to regulate the research, development, and manufacturing process of products toward commercialization [188]. Digital twin is an emerging vital technology for digital transformation and intelligent upgrades. It can perform functions such as monitoring, simulation, prediction, and optimization [189]. Digital twins can be applied to simulate and predict the process and results in vivo, which may accelerate clinical evaluation and translation.

Notably, antibody–drug conjugates (ADCs) have recently experienced a substantial resurgence, with the approval of multiple new ADC drugs and the progression of many additional ADC entities through various clinical development stages [190]. In an ADC, a bioactive molecule is tethered to an antibody with a designed linker. An ADC can target and deliver the attached compound to the disease-relevant cells/tissues while minimizing systemic toxicity. ABBV-3373 is a novel ADC composed of adalimumab linked to a proprietary and highly potent glucocorticoid receptor modulator (the anti-inflammatory payload) and is currently evaluated for RA treatment [191]. Inspired by this, The dual drug self-assembly co-delivery systems and peptide–drug conjugate nanomedicines may be promising in clinical translation.

Overall, further studies are required to extrapolate the data of nanotechnology-empowered combination therapy obtained through animal model studies to RA patients in clinical settings. More efforts are required to address the issues about safety, production, use, and end-life of nanoformulations, making them cost-effective, with a more rapid regulatory approval and successful market access.

## Conclusion and perspective

RA still remains a great challenge to cure due to its complexity and diversity, while pharmaceutical therapy dominates current clinical practice. To overcome the drawbacks of monotherapy and drug-related undesirable side effects, nano-empowered combination therapy has been extensively explored. On one hand, combination therapy is advantageous as exhibits synergistic pharmacological mechanisms with non-overlapping toxicities. On the other hand, by sharing expertise across fields, nanotechnology aids in developing advanced combination therapy through the co-delivery of therapeutic agents, exploitation of multifunctional nanomedicines, and exploration of multimodal therapy. This offers great promise in solving the bottleneck of current RA treatment in clinics either through formulation improvement or strategy innovation.

Regarding co-delivery, nanocarriers offer considerable variability in material choice to co-load therapeutic agents with improved physicochemical and biological properties. Most co-delivery strategies are currently developed empirically, and few studies have provided guidelines about combination regimens by using appropriate analytical methods. DDIs, unpredictable in vivo behaviors of coupled therapeutics, and the biological fate of nanoplatforms have made ensuring the efficiency and safety of co-delivery systems difficult. Accordingly, to gather informative data, save time, and achieve superior effects, the following steps are necessary: (1) Implementing artificial intelligence to boost the rational design. This includes screening bioinformatics and system maps of combined drug targets, predicting DDIs, and simulating the process in vivo. Data mining is a powerful approach based on the use of existing data for hypothesis testing and the development of predictive machine learning models [192], which may aid in searching for target interaction, DDIs, and prediction of biological responses of coupled therapeutics. Additionally, digital twins can monitor, simulate, and predict the process and result in vivo [193], possibly accelerating the clinical translation of co-delivery systems with predefined functionalities. (2) Conducting in-depth research on the mechanisms underlying different therapeutic agents from animal, cellular, and molecular levels. The interplay between nanomaterials and biological processes should also be investigated. Furthermore, it is important to verify whether the enhancement and reduction effect benefits from the simultaneously administered coupled therapeutics or the use of nanocarriers that optimize the distribution and enhanced retention in lesion sites. (3) Developing biosafe nanocarriers. Eco-friendly “green” nanocarriers and biomimetic nanocarriers are promising. The physiological and pharmacokinetic behaviors of nanocarriers in preclinical models should be studied to facilitate bridging to clinical trials. Moreover, protein corona may result in unpredictable biological outcomes by completely changing the properties of nanocarriers [194]. Therefore, the properties of nanoparticles and their interactions with the biological environment must be studied. (4) Carrying out additional work on the improvement of stability and on-demand release manner of nanocarriers. It is reasonable to expect the emergence of personalized co-delivery systems that could allow staged release of different therapeutics to specific disease microenvironments and pathogenic cells as the disease progresses. Furthermore, RA requires long-term medication, and advanced co-delivery platform-based wearable devices may offer real-time on-demand or preprogrammed self-administration to smartly manage different disease states.

Apart from serving as drug carriers, nanomaterials can extensively aid in RA treatment and diagnosis, thereby playing more than a supporting role in RA management. First, nanomaterials exhibit therapeutic effects owing to their special compositions or physicochemical properties. Nanomaterials with anti-inflammatory, oxidative stress regulation, and bone tissue repair properties have been widely explored. Nanomaterials with immune regulation and gene reprogramming functions must be further developed. In addition, nanomaterials that simultaneously serve as carriers and therapeutics support the theory of unifying medicines and excipients, which possibly have a greater clinical transformation ability. Second, nanomaterials can convert environmental stimuli and associated energy input into therapeutic effects. According to different energy conversion processes, treatment modalities can be classified as phototherapy, SDT, microwave therapy, and hydrogen-thermal therapy. How to integrate multiple modalities in a single nanoplatform and work simultaneously using a single excitation source needs to be determined. Only in this manner, the synergistic effects of drugs can be maximized, rather than the sequential use of different therapy modalities. Moreover, the related in-depth synergistic mechanisms between different modalities remain unelucidated. Third, nanomaterials allow rapid and accurate diagnosis of RA through non-invasive quantification of biological processes, which further facilitates the integration of diagnosis and treatment. Depending on the advancement of imaging and sensing technologies, nano-empowered combination therapy can be a closed-loop system allowing early diagnosis, personalized medical treatment, real-time monitoring of the treatment process, and provision of feedback on therapeutic effects. Nanorobotics offer advantages in imaging-guided diagnosis, drug delivery, therapy, and even surgery [195]. Nanorobotics that could enter remote sections of the body and perform diverse medical procedures are expected to emerge, bringing combination therapy into the age of the artificial intelligence industry.

In conclusion, based on the intricate etiology and pathogenesis of RA, as well as the current bottleneck associated with its treatment, this review elucidates the rationale and advantageous strategies of nano-empowered combination therapy. We emphasize on developing co-delivery systems, exploring therapeutical nanomaterials, and innovating in multimodal therapy. However, great challenges remain with RA treatment, including safety, DDIs, and clinical translation. When these issues are fully addressed, nano-empowered combination therapy will become widely applicable and available in clinics. Additionally, more efforts must be taken to develop a dynamic treatment targeting different stages of the disease in sequential phases by controlling the release of combination components. If possible, incorporating personalized treatment approaches would be highly beneficial. Moreover, the immune system must be re-engineered to halt the disease process before the occurrence of irreversible tissue damage. In the growing era of precision medicine, nano-based multimodal therapy integrating diagnosis and treatment plays a major role. Finally, we highlight the cross-innovation of different therapeutic agents, nanomedicines, and therapy modalities to benefit the treatment, rather than focusing on the theory of “the more the better.” Thus, choose your pattern carefully and utilize nanotechnology elaborately.

## Data Availability

No datasets were generated or analysed during the current study.
